# Red light excitation: illuminating photocatalysis in a new spectrum

**DOI:** 10.3762/bjoc.21.22

**Published:** 2025-02-07

**Authors:** Lucas Fortier, Corentin Lefebvre, Norbert Hoffmann

**Affiliations:** 1 Unité de Catalyse et de Chimie du Solide (UCCS), University of Lille, CNRS, University of Artois UMR 8181, Avenue Mendeleiev, 59655 Villeneuve d’Ascq CEDEX, France; 2 Laboratory of Glycochemistry and Agroressources of Amiens (LG2A), University of Picardie Jules Verne UR 7378, 10 rue Baudelocque, 80000 Amiens, France; 3 Institute of Physics and Chemistry of Materials of Strasbourg (IPCMS), University of Strasbourg UMR 7504, 23 rue du Loess, BP 43, 67034 Strasbourg CEDEX 2, France

**Keywords:** green chemistry, medicinal chemistry, organic photochemistry, photocatalysis, red-light mediated transformations

## Abstract

Red-light-activated photocatalysis has become a powerful approach for achieving sustainable chemical transformations, combining high efficiency with energy-saving, mild conditions. By harnessing the deeper penetration and selectivity of red and near-infrared light, this method minimizes the side reactions typical of higher-energy sources, making it particularly suited for large-scale applications. Recent advances highlight the unique advantages of both metal-based and metal-free catalysts under red-light irradiation, broadening the range of possible reactions, from selective oxidations to complex polymerizations. In biological contexts, red-light photocatalysis enables innovative applications in phototherapy and controlled drug release, exploiting its tissue penetration and low cytotoxicity. Together, these developments underscore the versatility and impact of red-light photocatalysis, positioning it as a cornerstone of green organic chemistry with significant potential in synthetic and biomedical fields.

## Introduction

Red-light-activated photocatalysis has recently gained significant interest as a tool for driving chemical transformations under mild and efficient conditions. The use of red and near-infrared light enables deeper penetration into reaction media, reducing the high-energy side reactions commonly triggered by UV or blue light. These features make red-light photocatalysis particularly advantageous for large-scale applications, offering enhanced safety and operational simplicity. Traditionally, research in this field has focused on metal-based photocatalysts, particularly those based on transition metals like ruthenium and osmium due to their intrinsic photophysical properties. However, with growing concerns around environmental sustainability, there is increasing interest in developing photocatalysts that are more accessible, tunable, and eco-friendly. Each section of this document discusses a specific approach to red-light photocatalysis, reflecting the field’s evolution and exploring diverse catalyst types and applications. The first section is dedicated to metal-based photocatalysts. Complexes involving metals such as osmium and ruthenium, have dominated red-light photoredox catalysis because of their ability to absorb low-energy photons and sustain redox cycles via stable excited states. In this section, the document highlights applications of these complexes in reactions like ring-closing metathesis and polymerization, where red light’s deeper penetration enhances yields and efficiency, particularly for large-scale reactions. The second section broadens the focus to explore organic photocatalysts. Unlike metal-based systems, organic photocatalysts such as phthalocyanins, squaraines and cyanins, offer effective electron and energy transfer under red-light irradiation without relying on transition metals. This shift towards organic catalysts opens new possibilities for sustainable photocatalysis, with applications ranging from selective oxidation to cross-dehydrogenative coupling. These organic systems are valued for their reduced environmental impact, their wide availability, and tunability, making them viable alternatives to traditional metal-based catalysts for red-light-driven transformations. The final section examines applications of red-light photocatalysis within biological and medicinal fields. The capacity of red light to penetrate biological tissues enables processes that are challenging or even impossible under UV or blue light. This section discusses different photocatalysts, such as helical carbenium ions and advanced nitrobenzofuran derivatives for applications in phototherapy and controlled drug release, underscoring the potential of red-light photocatalysis in biomedicine.

## Review

### Red-light photocatalysis with metal-based complexes

**Figure 1:**
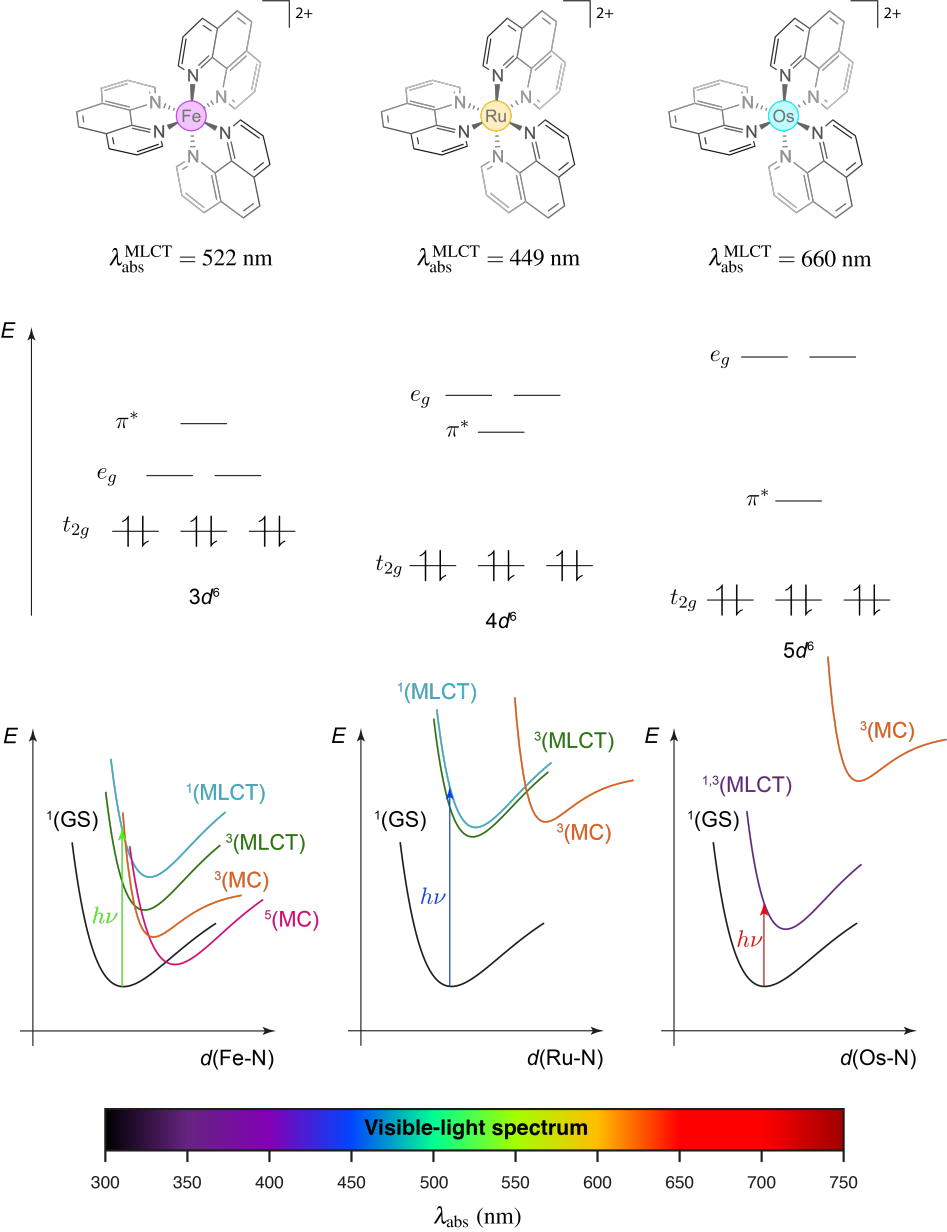
Influence of the metal center M (Fe, Ru, Os) on the position of the MLCT and MC (metal-centered) absorption band in the case of [M(phen)_3_]^2+^-type complexes (GS = ground-state).

Metal-based complexes naturally own a large span of colors depending on the nature of the metal and the ligands but also on the various oxidation states these compounds can attain. This property results on the absorption of a visible-light photon complementary to the observed color and has been extensively exploited in photoredox catalysis in recent years not only with heavy metals such as ruthenium and iridium [1–5], but also with lighter elements [6–8]. This field of light-mediated organic transformations relies on the use of a photocatalyst to promote radical reactions through electron transfer between this former and a given substrate or a sacrificial species. In the case of the metal-based complexes, the absorption band associated to the metal-to-ligand charge transfer (MLCT) is generally addressed even though other types of excitations like ligand-to-metal charge transfer, ligand- and metal-based excitation have been proven to be efficient in photoredox catalysis [9–12]. Actually, MLCT enables a charge separation for which the ligand-based electron can trigger a chemical reduction while the metal-centered hole, a chemical oxidation. This type of excitation is particularly enhanced in heavy metals, where the low-lying excited state often corresponds to the metal-to-ligand charge transfer (MLCT) transition. As the atomic number increases, relativistic effects become more pronounced, leading to the contraction of s and p orbitals while the d and f orbitals expand and become more diffuse. While these effects play a role in reducing the energy of the d orbitals and improving their overlap with the ligand orbitals, thereby facilitating electron transfer, the occurrence of metal-to-ligand charge transfer (MLCT) is also strongly influenced by other factors. Specifically, MLCT competes with metal-centered excitations (MC), which are governed by the ligand field splitting, an effect that increases for larger and more diffuse orbitals, and by the energy of the ligand’s π* orbitals. This interplay between relativistic effects, ligand field strength, and ligand orbital energy ultimately determines the absorption of lower-energy photons in second- and third-row transition metals compared to their first-row counterparts [13]. These phenomena are specifically improved when potent π-acceptor ligands are used due to their low-energy π* antibonding orbitals resulting in a bathochromic shift of the MLCT absorption band. These combined effects can be illustrated in the case of the [M(phen)_3_]^2+^ set with iron, ruthenium, and osmium (Figure 1). For a same phenanthroline ligand, these three complexes show an MLCT absorption band at different wavelengths, i.e., 522 nm for [Fe(phen)_3_]^2+^ [14], 449 nm for [Ru(phen)_3_]^2+^ and 660 nm for [Os(phen)_3_]^2+^ [15].

In a same way, spin–orbit coupling, stemming from the relativistic effects, can be exploited in photoredox-catalyzed reaction [16]. Spin–orbit coupling promotes intersystem crossing (ISC) between the singlet and triplet excited states, and even allows for direct excitation to the triplet state from the ground state *S*_0_. This effect mitigates rapid back-electron transfer from the singlet excited state to the ground state, extending the excited-state lifetime of the photocatalyst. Since the *T*_1_ → *S*_0_ transition is spin-forbidden, the process increases the overall efficiency of photoredox-catalyzed reactions.

**Scheme 1:**
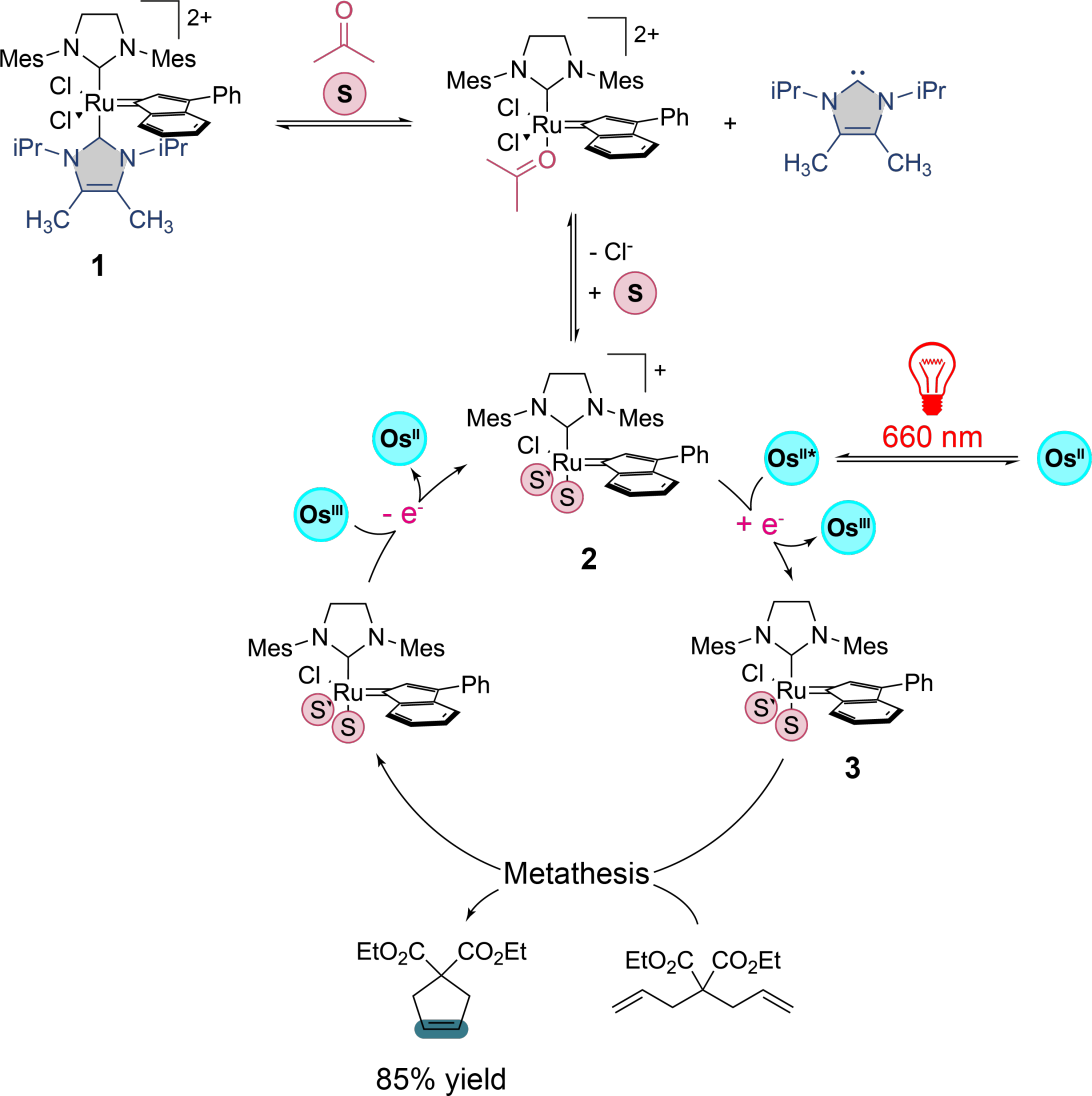
Red-light-mediated ring-closing metathesis through activation of a ruthenium catalyst by an osmium photocatalyst.

Absorption in the red region opens up innovative opportunities for photochemical transformations. First, the employed photon has the lowest energy in the electromagnetic spectrum of visible light, which allows for safer laboratory conditions in terms of irradiation apparatus, e.g., UV radiation sources. Secondly, from a synthetic perspective, a broader range of substrates and catalysts can be considered as in most cases, they are incapable of absorbing red light and, consequently, cannot initiate a photocatalytic transformation, thereby minimizing the risk of side reactions. This latter advantage has been notably exploited in the case of ring-closing olefin metathesis reactions, where Weizmann et al. utilized the photothermal response of plasmons from gold nanoparticles to activate the catalyst [17]. This approach contrasts with the work of Rovis et al. who employed a ruthenium(II) complex **1** activated through photoinduced electron transfer. The latter is pre-activated by the osmium complex shown in Scheme 1 after irradiation in the red region [18]. According to the mechanism proposed by the authors, the reaction is facilitated when conducted in acetone, allowing the pre-activation of the ruthenium complex with the successive release of an *N*-heterocyclic carbene ligand and a chlorine atom, which are replaced by two acetone molecules to form compound **2**. Simultaneously, excitation of the osmium(II) complex in the red region (660 nm) decreases its reduction potential to −0.97 V vs SCE, a value low enough to reduce the ruthenium complex **2**, whose potential is estimated at −0.89 V vs SCE, thereby yielding **3**, the active species for the metathesis reaction. The catalytic cycle is closed by the reduction of the resulting osmium(III) complex, regenerating the ruthenium(I) complex **2**.

**Scheme 2:**
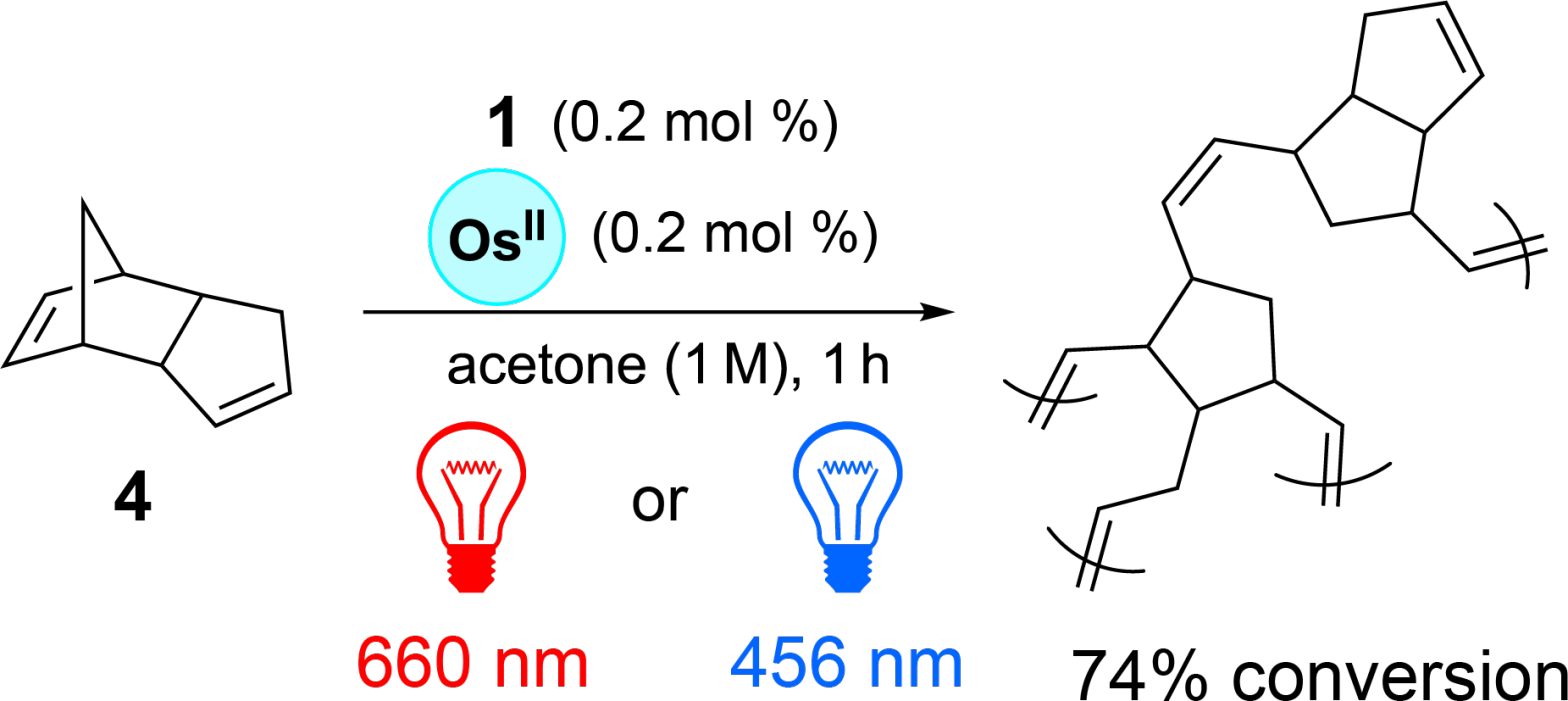
Photocatalyzed polymerization of dicylopentadiene mediated with red or blue light.

In this study, T. Rovis et al. demonstrated a third advantage of working with red light: its penetrating power. To this end, a polymerization reaction of dicyclopentadiene **4** was investigated through various materials such as amber glass, white paper, a solution of hemoglobin, and silicon by selectively irradiating a specific area of the reaction medium with red light (660 nm) or blue light (456 nm), respectively (Scheme 2).

**Figure 2:**
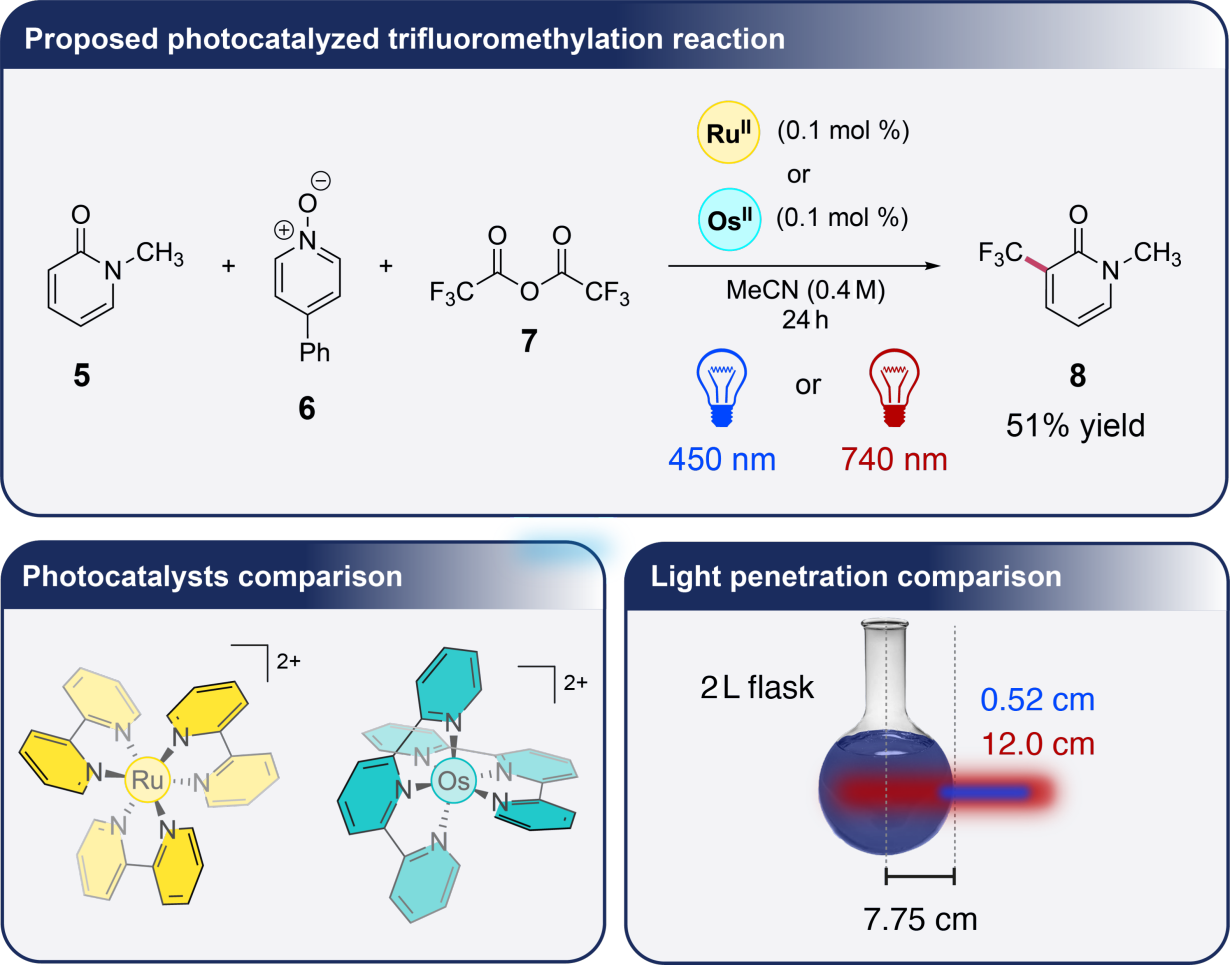
Comparison between [Ru(bpy)_3_]^2+^ and [Os(tpy)_2_]^2+^ in a photocatalyzed trifluoromethylation reaction: influence of the light source on the medium penetration. The flask in Figure 2 is taken from https://www.brusheezy.com, Free Photoshop Brushes by Brusheezy! This content is not subject to CC BY 4.0.

The authors have demonstrated that this polymerization reaction proceeded much more efficiently under red light irradiation. Indeed, red light can penetrate all the used materials and can initiate the reaction through the core of the reaction medium as the polymer forms, unlike blue light. This penetrating power of red light was also characterized by comparing the ruthenium complex [Ru(bpy)_3_]^2+^ absorbing at 450 nm with the osmium complex [Os(tpy)_2_]^2+^ absorbing at 740 nm in a the photocatalyzed trifluoromethylation reaction of **5** into **8** proposed by the same authors (Figure 2) [19]. These latter have shown the benefits of using their system based on an irradiation with red-light and the osmium complex over the ruthenium one with blue-light in a large-scale reaction. One of the most significant advantages is the superior light penetration of near-infrared light, which can reach deeper into the reaction mixture than blue light. In larger reaction volumes, where photon distribution becomes a limiting factor, near-infrared light penetrates approximately 23 times deeper than blue light when comparing the two molar extinction coefficients of [Os(tpy)_2_]^2+^ at 450 and 740 nm for a same concentration according to the Beer–Lambert law, hence ensuring more uniform photon exposure and an improved reaction efficiency. This enhanced light penetration is reflected in the reaction yields as a function of the reaction-scale. As this latter increases, the obtained yields using [Ru(bpy)_3_]^2+^ under blue light decrease significantly to reach a yield loss of 31.6% at a 250× scale, while those using [Os(tpy)_2_]^2+^ under near-infrared light remain constant or even increase up to a yield gain of 27.5% at the same reaction scale. T. Rovis et al. emphasize this result has profound implications for industrial applications. The ability of red light to penetrate deeply and to maintain high efficiency without requiring specialized flow reactors or high-powered light sources makes the [Os(tpy)_2_]^2+^/red-light irradiation system well-suited for large-scale manufacturing. In this way, the authors have also tested different osmium complexes in various well-established photocatalyzed reactions such as copper, palladium, cobalt, and nickel metallophotoredox couplings using red light, thereupon highlighting potential for broad applications in photoredox catalysis on an industrial scale.

**Scheme 3:**
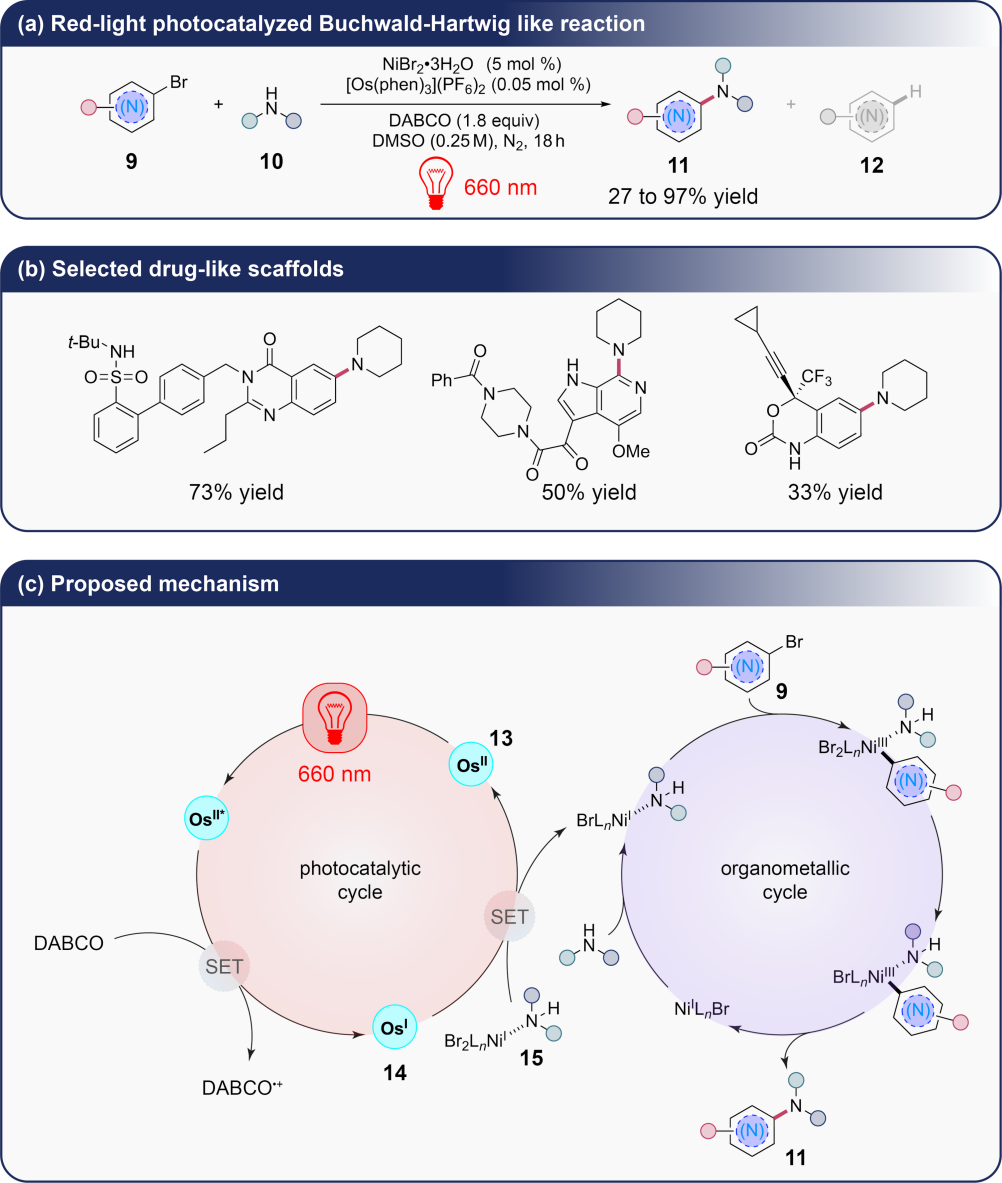
Red-light photocatalyzed C–N cross-coupling reaction by T. Rovis et al*.* (SET = single-electron transfer).

In this context, T. Rovis et al. have studied a C–N cross-coupling Buchwald–Hartwig-like reaction using dual nickel and osmium catalysis under red-light activation, addressing common challenges such as poor light penetration, side reactions, and scalability related to traditional blue-light-driven metallophotoredox reactions. By switching from blue light (460 nm) to red light (660 nm) and osmium complex [Os(phen)_3_]^2+^ instead of an iridium complex, the authors have been able to significantly enhance the scope and efficiency of these reactions. Specifically, they demonstrate that red light can suppress unwanted side reactions, such as hydrodehalogenation (compounds **12**, Scheme 3a), a common issue in high-energy light systems where aryl–Ni bonds are cleaved, leading to undesirable byproducts. The study found that by using [Os(phen)_3_]^2+^ as the photocatalyst and 660 nm red light, the reaction exhibited greater functional group tolerance, handling a variety of electron-deficient, neutral and rich (hetero)aryl bromides **9** and primary and secondary amine-based nucleophiles **10** with minimal degradation or side reactivity in a scope of around 50 examples with yields ranging from 27 to 97 % compared to the use of blue-light (Scheme 3a) [20]. In this way, T. Rovis et al. have exploited their photocatalytic system on the functionalization of drug-like scaffolds with moderate to good yields (Scheme 3b). The mechanism of the reaction presented by the authors involves two different catalytic cycles as presented in Scheme 3c. After excitation of the osmium complex **13**, this latter is reduced via the use of a tertiary amine to give the active species **14** able to oxidize the formed nickel complex **15** in the reaction mixture. This step allows the oxidative addition of nickel on the aryl bromide **9** followed by the reductive elimination giving the desired product **11**. Besides the innovative synthetic results obtained in this study, the authors underline a major advantage to switch to red light as it enables a deeper penetration through the reaction media, making the method highly scalable. In a batch reaction, the authors have successfully scaled up the synthesis by two orders of magnitude, achieving comparable yields without the need for complex flow reactors.

**Figure 3:**
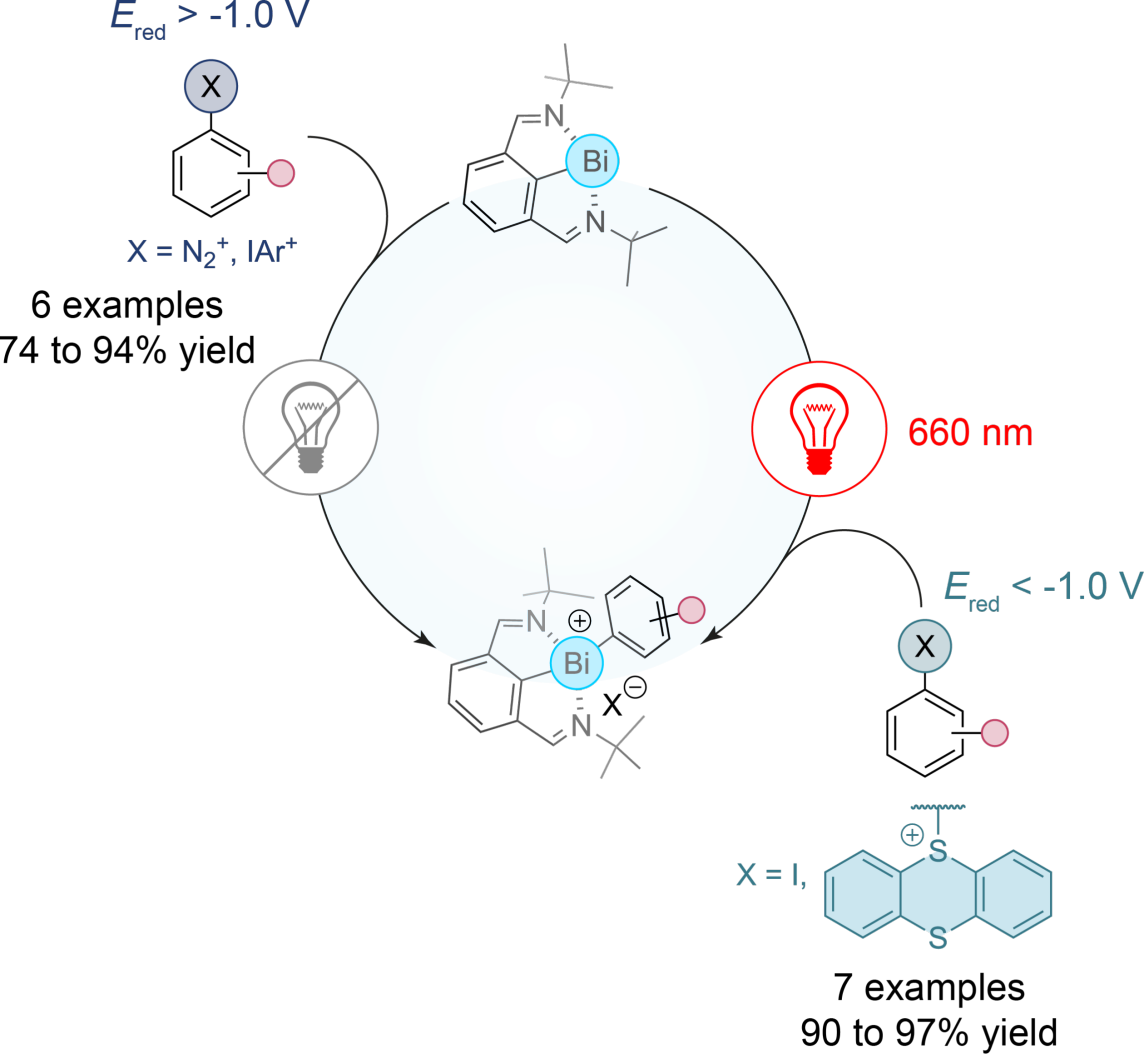
Red-light-mediated aryl oxidative addition with a bismuthinidene complex.

The successful implementation of osmium complexes in large-scale photoredox reactions highlights the broader applicability of transition metals in facilitating challenging chemical transformations. Moving beyond the d-block, the exploration of main-group elements offers a new frontier in the use of more affordable and available photocatalysts. While transition metals such as copper, palladium, cobalt, and nickel are well-established in catalyzed cross-coupling reactions, J. Cornella et al. have highlighted the reactivity of main-group elements like bismuth, which can mimic transition-metal behavior through oxidative addition. In their recent study, the authors have developed a complementary ground-state- and excited-state-driven aryl oxidative addition platform based on an *N*,*C*,*N*-bismuthinidene complex, showing the unique capacity of this main-group element to engage in reactivity typically associated with d-block metals [21]. The study explores how this bismuth(I) complex undergoes oxidative addition with a variety of aryl electrophiles, including diazonium salts, iodonium salts, and challenging aryl iodides and aryl thianthrenium salts, typically requiring transition-metal catalysts (Figure 3). The reactivity of the *N*,*C*,*N*-bismuthinidene complex is made possible through both ground-state and photoexcited-state processes, where bismuth's ability to access multiple oxidation states facilitates the formation of stable aryl–bismuthonium complexes. Notably, the authors have demonstrated that by harnessing low-energy red light, the *N*,*C*,*N*-bismuthinidene complex can drive formal oxidative addition even with substrates that exhibit high reduction potentials such as aryl iodides and aryl thianthrenium salts, thereby expanding the scope of aryl electrophiles that can be subjected to oxidative addition. This mechanistic advancement, combined with the ability to operate under low-energy light conditions, opens up new avenues for main-group redox catalysis in organic synthesis. By leveraging light excitation to enhance the reducing power of the bismuth complex, the study showcases the potential of main-group photoredox systems to complement traditional transition-metal catalysis. As the use of osmium catalysts has already demonstrated scalability in industrial applications [22], the introduction of bismuthinidene complexes presents another step forward in expanding the photoredox catalysis toolkit, potentially paving the way for more sustainable and efficient catalytic processes.

**Scheme 4:**
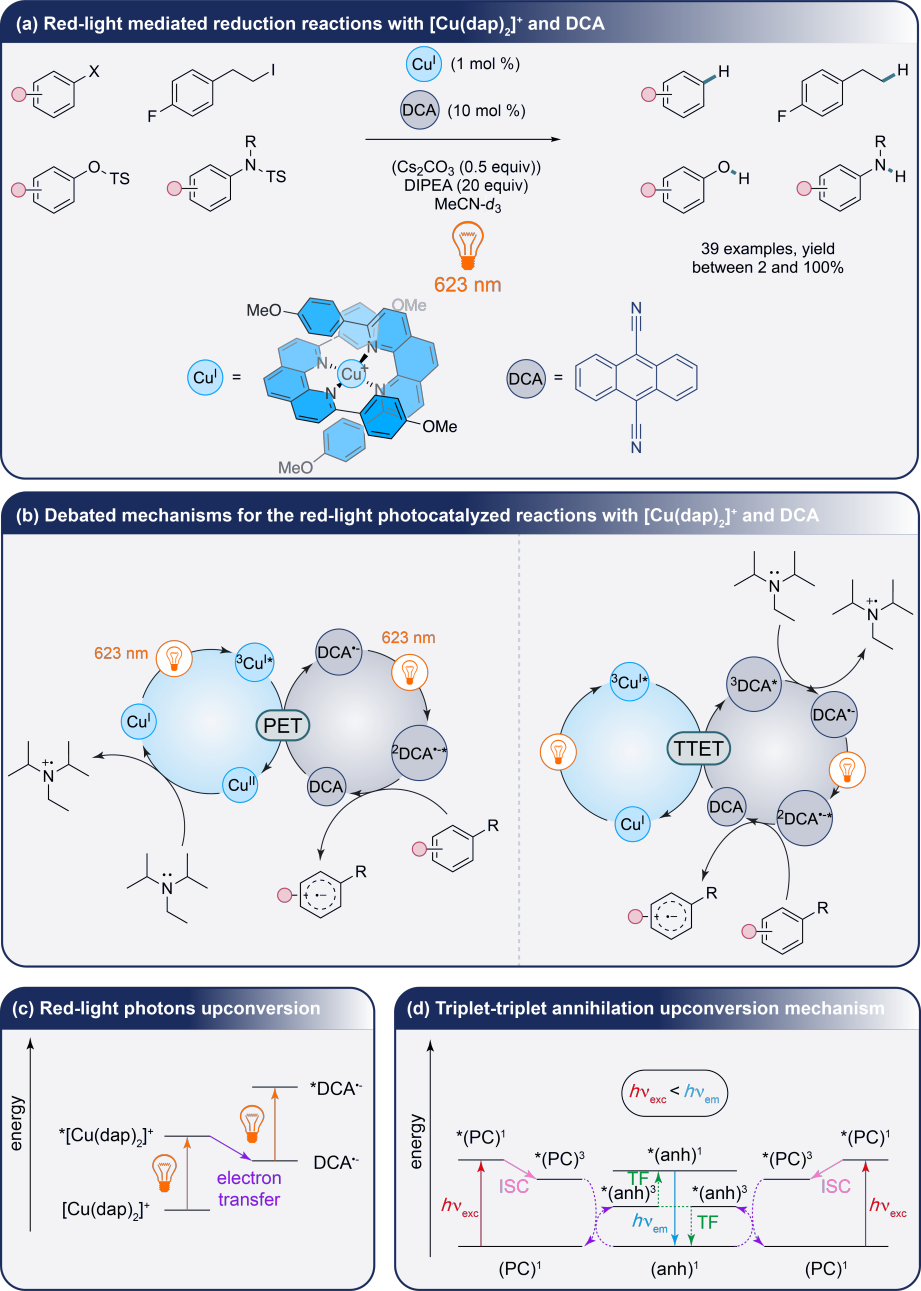
Red-light-mediated reduction of aryl derivatives by O. S. Wenger et al. (PC = photocatalyst, anh = annihilator, ISC = intersystem crossing, and TF = triplet fusion).

In the same way, recent advances in red-light photoredox catalysis have expanded the utility of first-row transition metals in a domain traditionally dominated by second- and third-row elements. A prime example of this evolution is the development of a dual photoredox strategy, which exploits the properties of red-light excitation while addressing the energy constraints posed by low-energy photons. Whereas multiple works addressing the upconversion phenomenon involving heavy metals like palladium, platinum [23–24] or osmium [25], grafted ruthenium complexes over ytterbium and thulium nanoparticles [26] and the use of a molybdenum-centered [27] and tungsten-centered complexes have been described in the literature [28]. The work by O. S. Wenger et al. introduces a system that mimics the Z-scheme of photosynthesis, utilizing a copper(I) bis(α-diimine) complex in combination with 9,10-dicyanoanthracene radical anion (DCA^•−^) [29]. This system effectively drives photoredox-mediated reduction and C–C cross-coupling reactions under mild red-light conditions with various aryl halides, an aliphatic iodide, and *O*- and *N*-tosylated substrates (Scheme 4a). The authors discuss two plausible mechanisms: a photoinduced electron transfer (PET) between the ^3^MLCT state of the copper complex and DCA or a triplet–triplet energy transfer (TTET) between the ^3^MLCT state of the copper complex and DCA. In both of these mechanisms, the involvement of a doublet excited-state of the radical anion of the DCA, photodegraded species issued from DCA or DCA^•−^/substract donor–acceptor complex is debated (Scheme 4b). Nevertheless, the authors underline that the key feature of these transformations is going through the consecutive absorption of two red-light photons which enhances the photoredox process by converting these latter into a higher-energy excited state (Scheme 4c) unlike what has been done until now with various described systems already discussed in the literature and emission of blue-light in the case of the triplet–triplet annihilation upconversion phenomenon among others (Scheme 4d) [30].

**Scheme 5:**
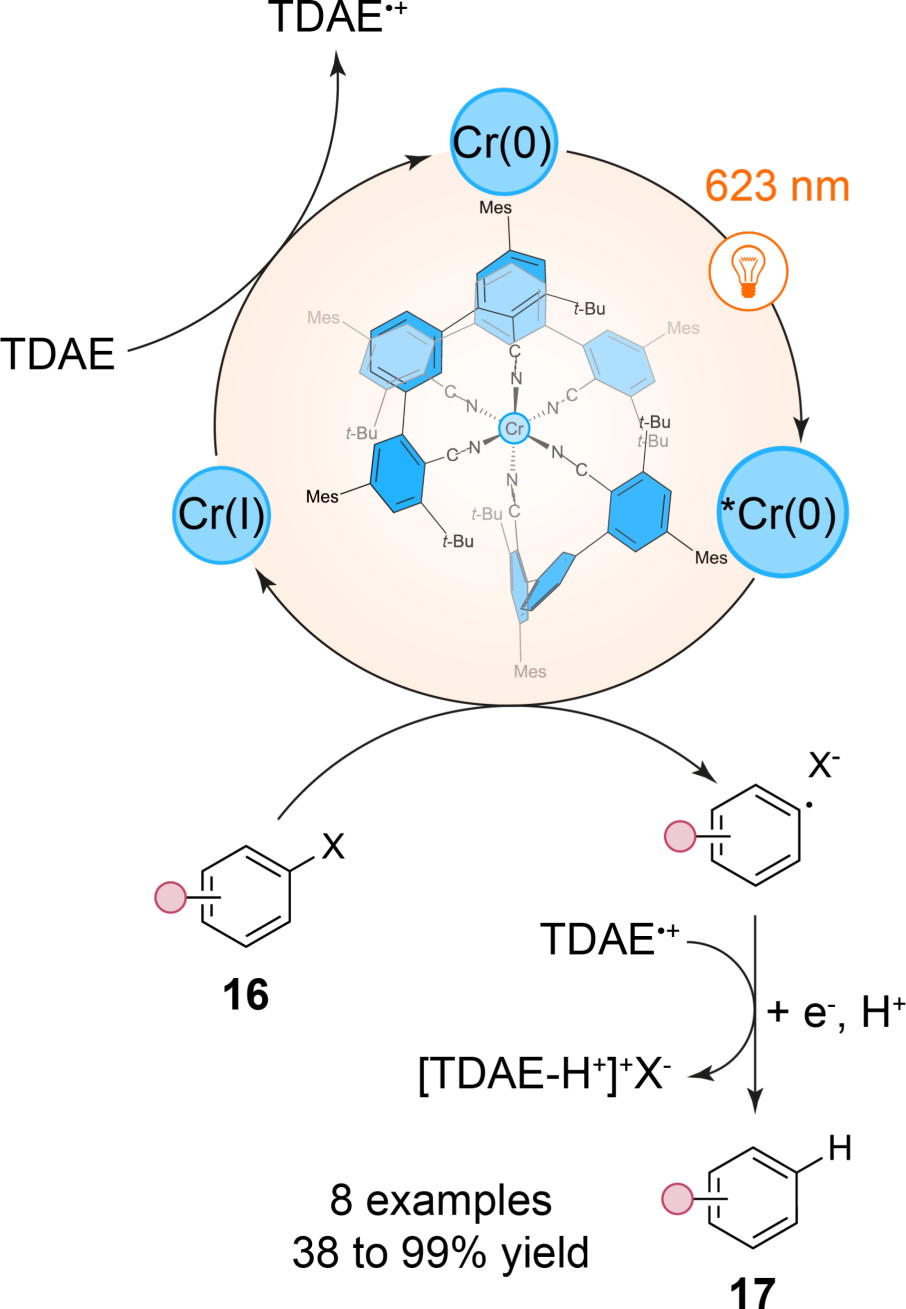
Red-light-mediated aryl halides reduction with an isoelectronic chromium complex (TDAE = tetrakis(dimethylamino)ethylene).

In a continuation of this trend, a recent study by O. S. Wenger et al. further expands the frontiers of red-light photoredox catalysis with first-row transition metals by introducing Cr(0) luminophores that exhibit photophysical properties competitive with Ru(II) and Os(II) complexes [31]. These new isoelectronic tris(diisocyanide)Cr(0) complexes, [Cr(L^Mes^)_3_] (Mes = mesityl) and [Cr(L^Pyr^)_3_] (Pyr = pyrenyl), display remarkably high metal-to-ligand charge transfer (MLCT) lifetimes of up to 47 ns and photoluminescence quantum yields as high as 1.04%. This surpasses previously reported first-row d^6^ metal complexes such as Fe(II) complexes and positions Cr(0) as a promising alternative in photoredox applications under low-energy red-light conditions. The enhanced performance of these Cr(0) complexes is attributed to their strong ligand field, provided by the isocyanide ligands, which raises the energy of metal-centered states and minimizes non-radiative decay pathways. This design results in prolonged MLCT excited states, making these complexes suitable for challenging triplet–triplet energy transfer (TTET) and photoredox catalysis reactions such as hydrodehalogenation of aryl iodides, bromides and even chlorides **16** into **17** (Scheme 5).

This shift towards sustainable photocatalysis with first-row transition metals has been further emphasized by the work of O. S. Wenger et al. who has introduced another isoelectronic Cr(0) d^6^ metal complex capable of sensitized triplet–triplet annihilation upconversion. This system achieves red-to-blue upconversion under red-light irradiation, rivaling the performance of traditional heavy-metal systems such as Os(II) complexes. By employing a Cr(0) photosensitizer combined with a silylacetylene-decorated anthracene annihilator, the authors have reported an upconversion efficiency of 1.8% and have demonstrated its utility in initiating blue light-dependent polymerization reactions under red-light conditions [32].

**Scheme 6:**
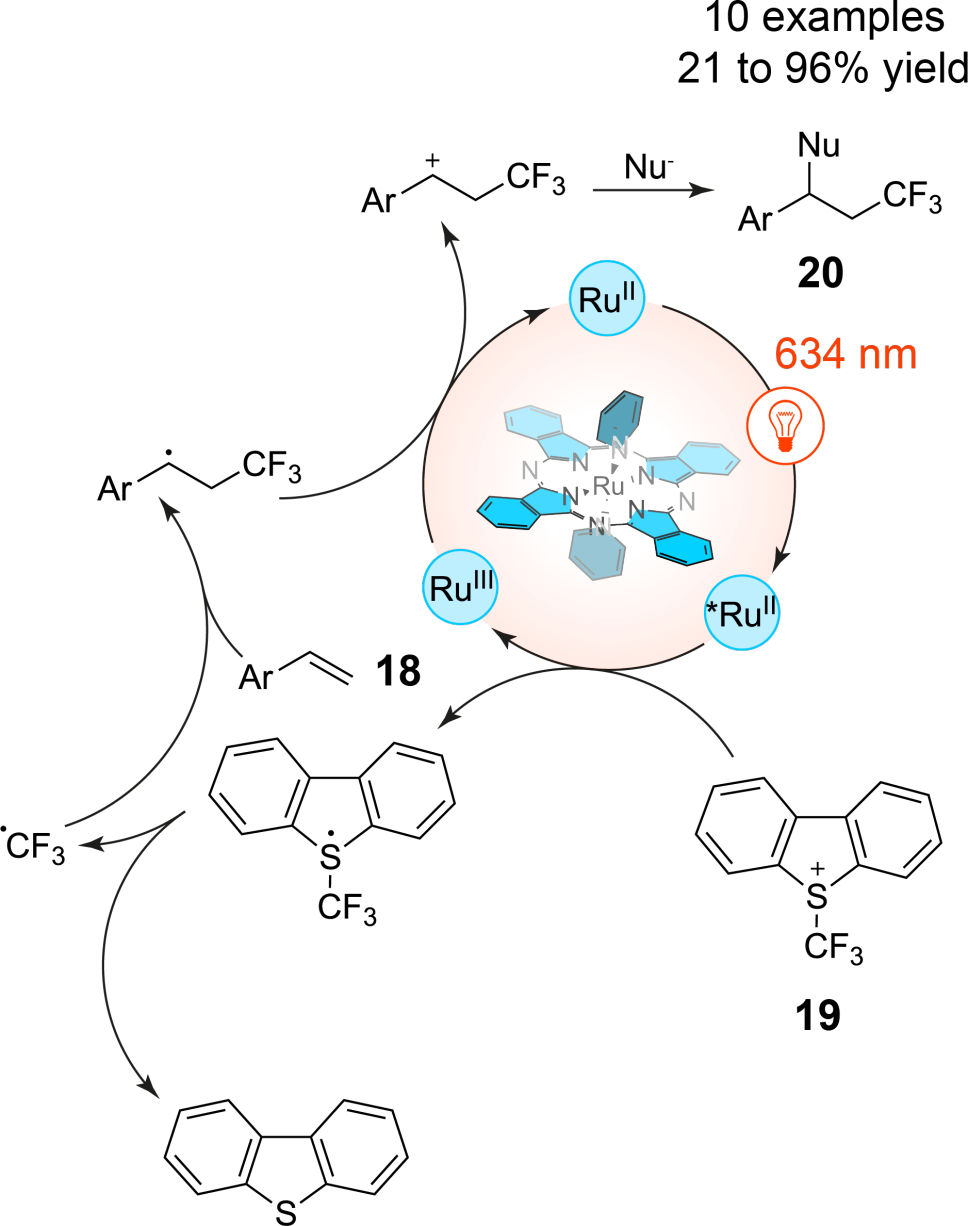
Red-light-photocatalyzed trifluoromethylation of styrene derivatives with Umemoto’s reagent and a phthalocyanin ruthenium complex.

Beyond the position of transition metals in the periodic table, ligand design plays a crucial role in determining the photophysical properties of metal complexes. Phthalocyanins, porphyrins, and their derivatives exemplify this, as their rigid macrocyclic structures enable strong absorption in the visible to NIR regions, making them appealing for photoredox catalysis applications. For instance, ruthenium phthalocyanin complexes have emerged as potent catalysts for red-light-mediated photoreactions. Furuyama et al. demonstrated that a ruthenium phthalocyanin complex could catalyze trifluoromethylation reactions of styrene derivatives **18** with either CF_3_SO_2_Cl or Umemoto’s reagent **19** under red-light irradiation without the need for sacrificial reducing agents (Scheme 6), contrasting with traditional blue-light photocatalysis which led to substrate decomposition [33–34]. The axial ligands on the ruthenium phthalocyanin complex, particularly electron-deficient pyridyl groups, were found to influence the catalytic activity by stabilizing the excited states and promoting metal-to-ligand charge transfer (MLCT) pathways, which is critical for efficient photoreactivity.

**Scheme 7:**
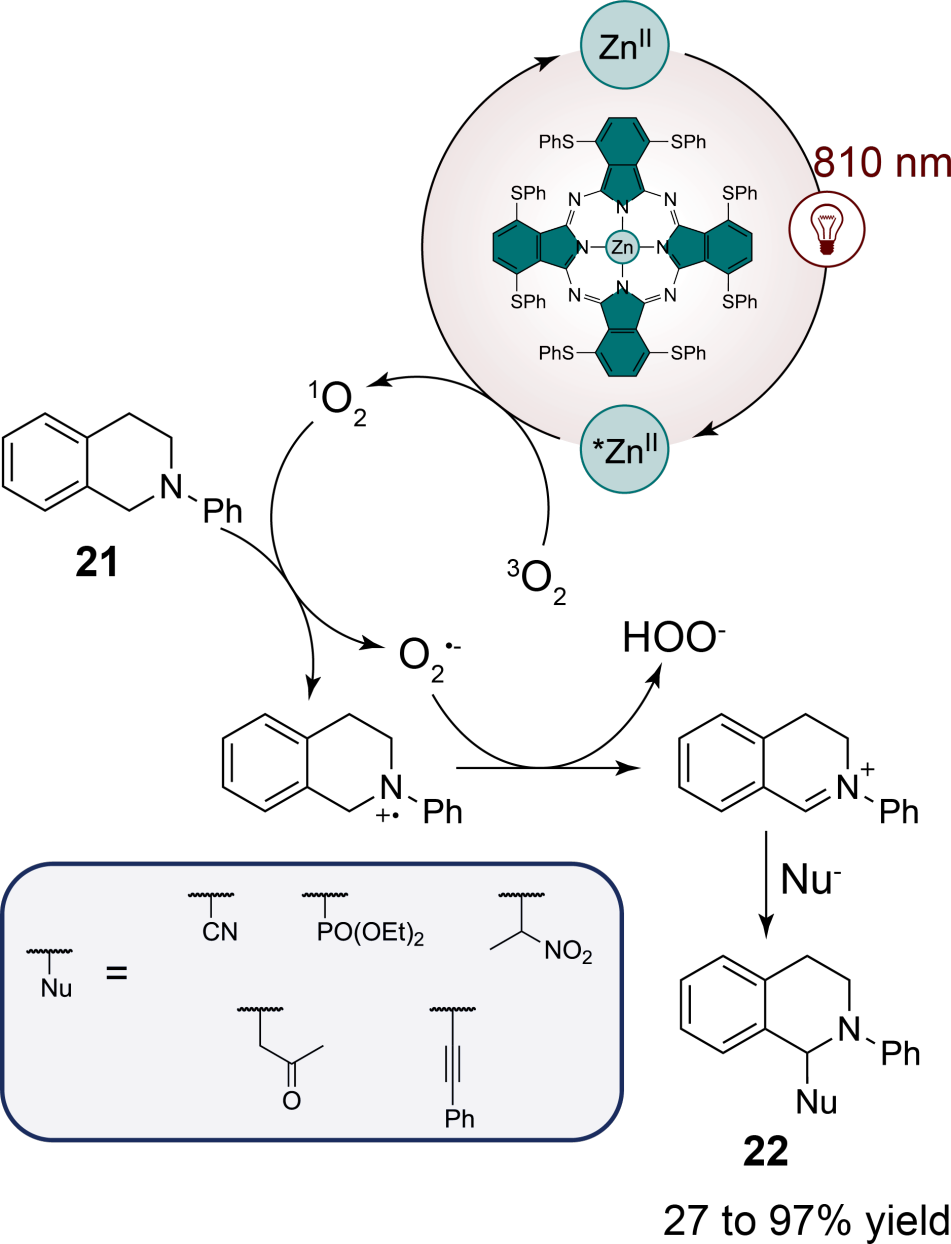
Red-light-mediated energy transfer for the cross-dehydrogenative coupling of *N*-phenyltetrahydroisoquinoline with nucleophiles.

Interestingly, phthalocyanin ligands are not only effective with precious metals but also exhibit catalytic performance when coordinated with first-row transition metals such as zinc as proposed by Furuyama et al. [35]. In this way, the authors have developed a series of phthalocyanin zinc complexes with electron-rich sulfur atoms at the non-peripheral positions of the phthalocyanin ring. This functionalization allows the destabilization of the highest occupied molecular orbital (HOMO), thereby shifting the absorption of the complexes into the NIR region (around 810 nm). The authors have demonstrated the efficiency of their photocatalyst in cross-dehydrogenative coupling reactions with *N*-phenyltetrahydroisoquinoline **21** and diverse nucleophiles (Scheme 7). Their photocatalyst has shown superior yields compared to free-base and other phthalocyanin first-row transition-metal complexes, such as nickel. The choice of solvent was also critical for optimizing the reaction efficiency: a mixture of pyridine and methanol not only improved the yield but also has helped to suppress side reactions. The cross-dehydrogenative coupling reactions, under near-infrared irradiation, was found to proceed via an energy-transfer mechanism involving singlet oxygen generation rather than the typical electron-transfer pathway observed in the presented visible-light-mediated reactions in this review hitherto. This singlet oxygen is generated by the energy transfer from the excited state of the phthalocyanin zinc complexes to molecular oxygen, allowing the oxidation of the *N*-phenyltetrahydroisoquinoline **21** into a reactive iminium intermediate that subsequently couples with nucleophiles to give **22**.

**Scheme 8:**
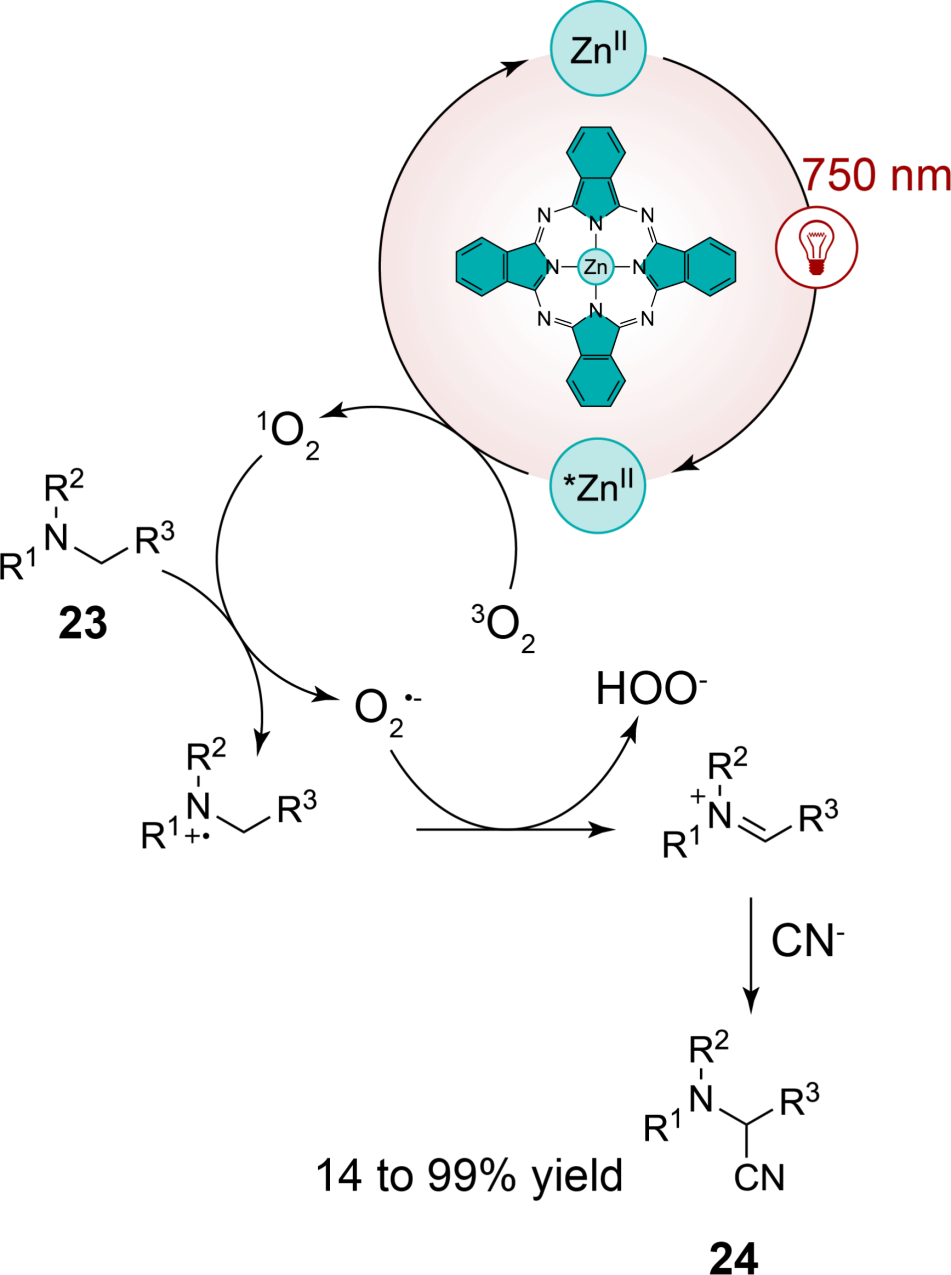
Red-light-mediated oxidative cyanation of tertiary amines with a phthalocyanin zinc complex.

In a same manner, Opatz et al. have shown that zinc phthalocyanins can catalyze oxidative cyanation reactions of tertiary amines **23**, yielding α-aminonitriles **24** under continuous-flow conditions [36]. This reaction proceeds through the excitation of zinc phthalocyanin by near-infrared light (λ = 750 nm), followed by triplet energy transfer to molecular oxygen, generating singlet oxygen as the active species. Similarly as in the case of the Furuyama et al. study, the singlet oxygen subsequently oxidizes the amine substrate to an iminium ion, which reacts with a cyanide nucleophile to form the desired α-aminonitrile (Scheme 8). Notably, the authors have optimized the reaction conditions to achieve high yields across a wide substrate scope with more than 15 examples, including the cyanation of aliphatic amines such as tributylamine and sterically hindered substrates, which demonstrated the broad applicability of this photocatalytic system.

**Scheme 9:**
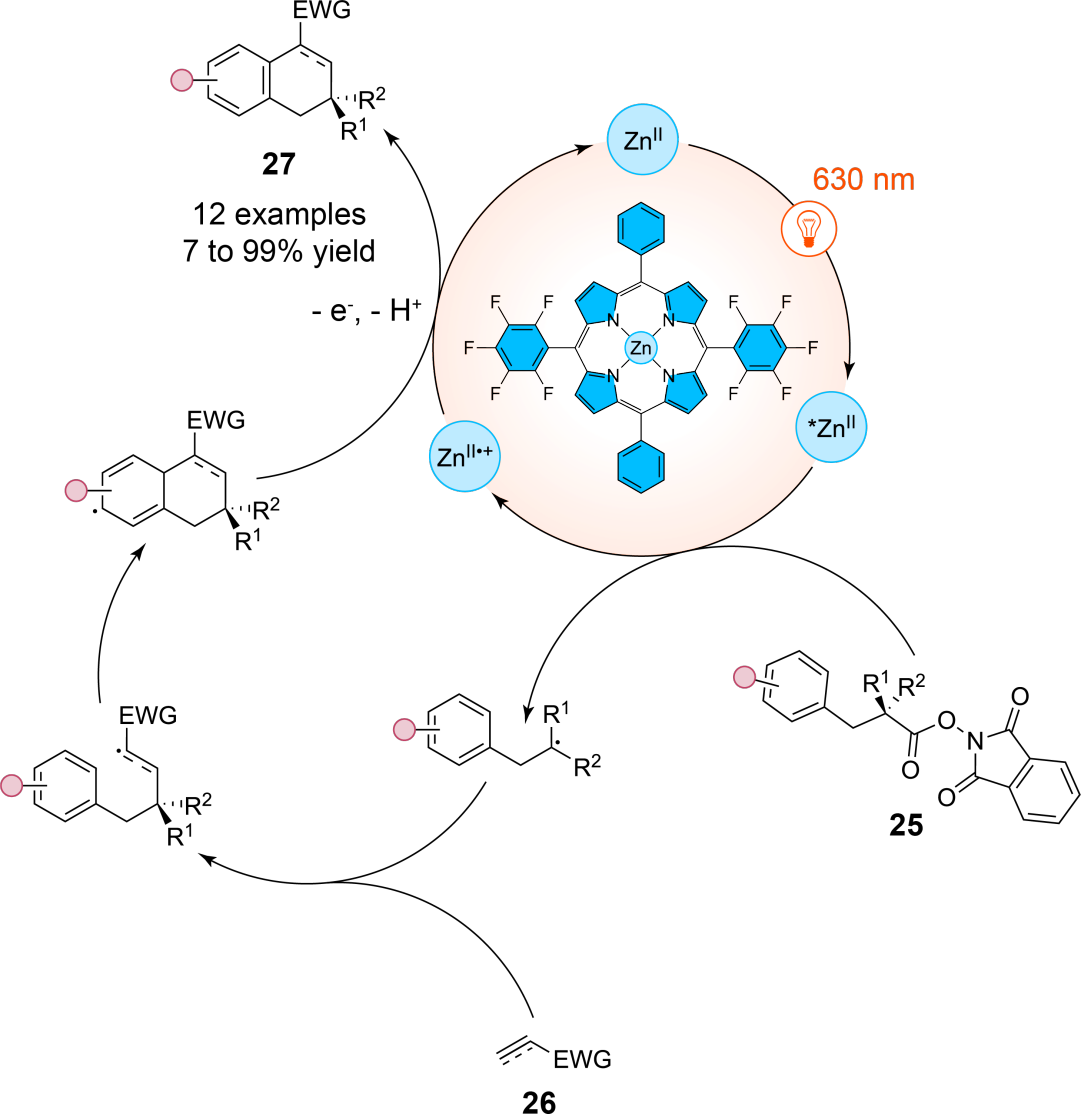
Formation of dialins and tetralins via a red-light-photocatalyzed reductive decarboxylation mediated by a zinc(II) porphyrin complex.

It has to be noted that zinc-based photocatalysts with phthalocyanin ligands are not limited to energy-transfer pathways involving singlet oxygen generation. A recent work by Yoshimitsu et al. has shown that zinc(II)-based porphyrin complexes can engage in electron-transfer mechanisms to enable radical cascade reactions under red-light irradiation (Scheme 9) [37]. These zinc(II)-based porphyrin catalysts operate via an oxidative quenching cycle, directly facilitating the transfer of an electron from the excited state of the porphyrin to the substrate, an activated ester **25**, subsequently generating carbon-centered radicals without the need for sacrificial electron donors via a decarboxylation process. In reacting with electron-deficient alkenes or alkynes **26**, these radicals further yield tetralin and dialin moieties **27**, respectively. Among their scope of 26 examples with yields ranging from 7 to 99%, the authors have shown that the use of dimethyl fumarate as a radicophile yielded the desired tetralin in 99% yield, while other radicophiles such as ethyl propiolate also furnished dialin products in high yields mainly superior to 70%.

**Scheme 10:**
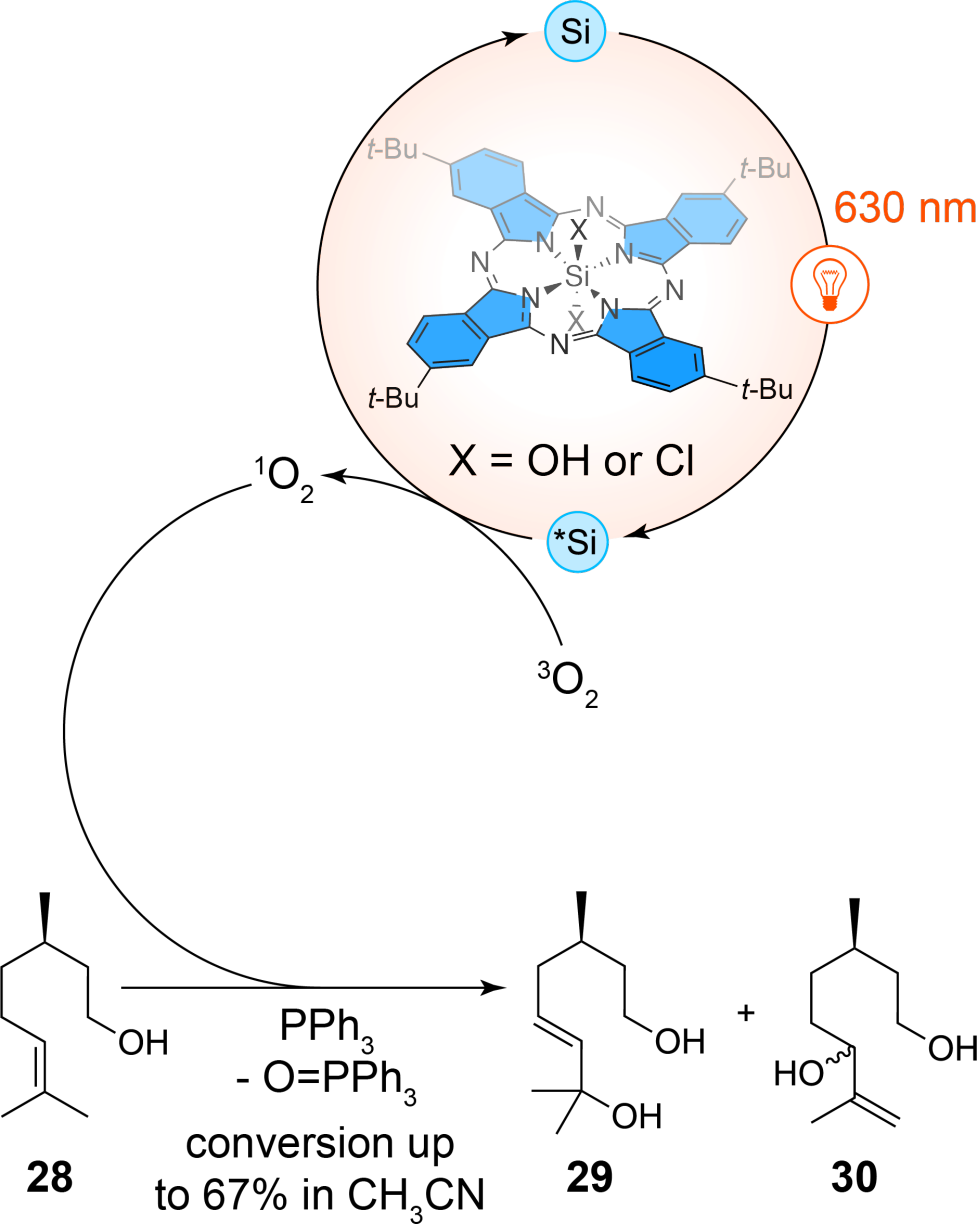
Oxidation of β-citronellol (**28**) via energy transfer mediated by a red-light activable silicon phthalocyanin complex.

Beyond their coordination with transition metals, phthalocyanin ligands also exhibit remarkable photoreactivity in supporting non-metallic central elements. Recent work by Amara et al. highlights the efficiency of silicon-based phthalocyanin complexes in red-light-driven photooxidation processes, marking a shift away from the reliance on traditional heavy-metal catalysts [38]. By utilizing silicon as a cheap, non-toxic, and abundantly available element, silicon-based phthalocyanin complex derivatives represent a more sustainable alternative to precious metal-based photocatalysts such as ruthenium or palladium complexes. The authors have focused on the photooxidation of β-citronellol (**28**), a key step in the production of industrial compounds like rose oxide (Scheme 10). Remarkably, the silicon phthalocyanins demonstrated exceptional stability under red-light irradiation, achieving up to 87% conversion in continuous-flow conditions. This performance is particularly notable given that the reaction was carried out using sub-part-per-million loadings of the catalyst (0.003 mol %), a stark contrast to traditional systems, which often require higher concentrations of heavy metals. Unlike classical transition-metal-based photosensitizers acting by triplet energy transfer for which high singlet oxygen quantum yields are crucial, the silicon phthalocyanins in this study have exhibited relatively low singlet oxygen quantum yield values (around 0.27 for the silicon-based photocatalysts used in this work). Despite this, their catalytic performance was not compromised. The key to their success lies in their resistance to photobleaching, which is a common issue for metal-based photocatalysts that degrade under prolonged exposure to light. The silicon-based phthalocyanin complexes maintained their structural integrity over extended reaction times, even under high substrate concentrations and continuous flow conditions, achieving high turnover numbers of over 50000. This stability allowed for solvent-free reactions, significantly enhancing the sustainability of the process. Additionally, the authors have explored the scalability of these systems, demonstrating their efficacy in multigram-scale photooxidations. The use of silicon-based phthalocyanin complexes in continuous-flow reactors not only increased the productivity of β-citronellol oxidation but also reduced the process mass intensity by a factor of four compared to batch processes using conventional solvents.

**Scheme 11:**
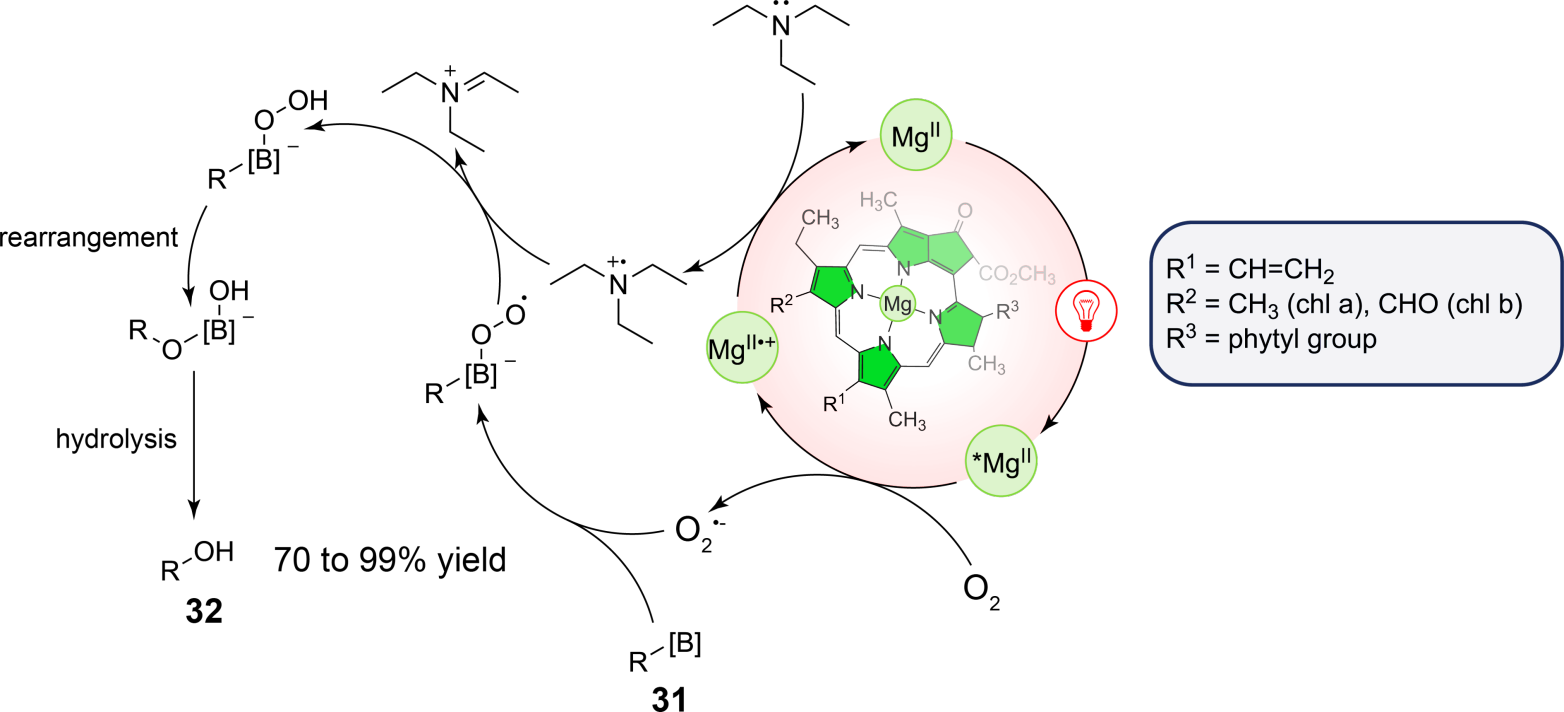
Formation of alcohol derivatives **32** from boron compounds **31** using chlorophyll (chl) as a red-light-absorbing photocatalyst.

Recent advances have demonstrated that naturally derived photocatalysts, such as chlorophyll, a magnesium-based chlorin complex, can also efficiently drive photocatalytic transformations. In the study by Ouyang et al. chlorophyll extracted from spinach has been employed as a green and sustainable photocatalyst for the red-light-induced oxidation of organoboron compounds, a method that stands out for its environmental and operational simplicity [39]. The authors have developed a mild protocol utilizing red light to oxidize a wide range of organoboron substrates **31**, including -B(OH)_2_, -Bpin, -BF_3_K, and -Bneo (neopentyl borate) derivatives noted as “-[B]” in Scheme 11, to produce aliphatic alcohols and phenols **32** with moderate to excellent yields. The versatility of this method is highlighted by the broad substrate scope of more than 50 examples, which includes various aryl- and alkylboronates, showcasing its applicability across a range of chemical transformations. For example, phenylboronic acids with diverse substitution patterns (such as electron-donating and electron-withdrawing groups) were smoothly converted to the corresponding alcohols in yields ranging from 70% to 99%, with minimal byproducts. Even more complex substrates, like naphthalene and pyrene derivatives, underwent efficient oxidation, suggesting the method’s utility in functionalizing more challenging aromatic frameworks. The mechanism of this reaction was explored in detail, and the authors have proposed that the reaction proceeds through a photoinduced electron transfer mechanism (Scheme 11). Upon red-light excitation, chlorophyll generates superoxide anion radicals (O_2_^•−^) in the presence of oxygen, which act as the active oxidant to convert organoborons **31** to alcohols **32**. Notably, this protocol is both energy-efficient and operationally simple, with the reaction being carried out under air in ethanol, a low-toxicity solvent. Moreover, the scalability of the reaction was demonstrated with larger-scale experiments (up to 5 mmol), achieving excellent yields and minimal waste. The authors further showcased the robustness of the method by utilizing common kitchen equipment, mixing spinach extract with Baijiu (a traditional Chinese drink with a high level of ethanol) and stirring the reaction under sunlight, achieving yields as high as 60%. This demonstration underscores the practicality and accessibility of the method, even outside a laboratory setting. The green chemistry metrics analysis provided in the study confirms the environmental benefits, with high atom economy, low waste generation, minimal resource consumption, and the potential of porphyrin/phthalocyanin derivatives as ligand of high interest in red-light-mediated photocatalyzed transformations.

### Red-light photocatalysis with organic molecules

**Scheme 12:**
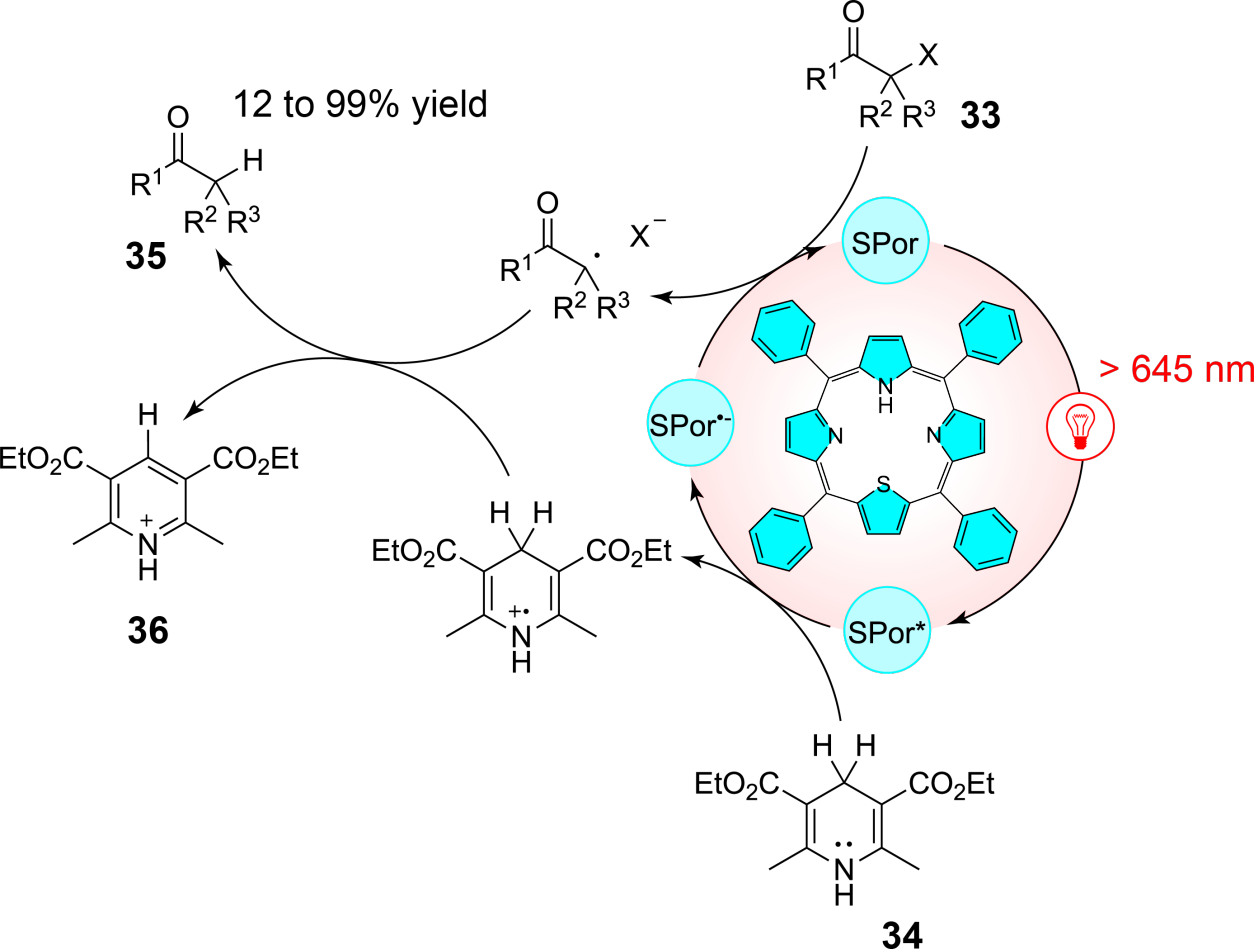
Red-light-driven reductive dehalogenation of α-halo ketones mediated by a thiaporphyrin photocatalyst.

While the use of metal-based porphyrin/phthalocyanin complexes has proven their efficiency in red-light-driven chemical transformations, it has to be noted that the photocatalytic efficiency of these molecules does not necessarily rely on the presence of a central metal atom. Free-base porphyrins, in particular, have demonstrated significant potential in red-light-driven transformations due to their versatile photophysical properties. These metal-free systems can function as both photooxidants and photoreductants, engaging in either energy-transfer or electron-transfer processes with high efficiency [40]. Notably, D. Gryko et al. demonstrated their application in activating diazoalkanes through red-light-mediated photosensitization and photoredox catalysis for carbene transfer reactions and radical-based functionalizations [41]. In another study by Derksen et al., thiaporphyrins were introduced as highly effective catalysts for red-light-mediated photoreductive dehalogenation [42]. These thiophene-modified porphyrins exhibit excellent absorption properties beyond 645 nm, providing access to low-energy red-light-driven chemical transformations. The catalysts have successfully facilitated the dehalogenation of 18 different α-halo ketones **33** with minimal catalyst loading (0.1 mol %) and high yields under mild conditions. For instance, bromoacetophenone has been quantitatively reduced to the corresponding methyl ketone within one hour under red-light irradiation. The optimized reaction conditions proposed by the authors have demonstrated the superiority of thiaporphyrins over conventional metal-based systems like Ru(bpy)_3_Cl_2_. When tested, one of the thiaporphyrins has achieved significantly higher yields (75%) compared to the Ru-based photocatalyst, which has only afforded a modest 18% yield under similar conditions. This enhanced reactivity was attributed to the strong reduction potential of the thiaporphyrin catalysts and their ability to participate in hydrogen-atom-transfer mechanisms with a Hantzsch ester **34** as presented in Scheme 12. Moreover, the study has explored the impact of substrate steric hindrance and halogen bond strength on catalytic efficiency, revealing that bromo- and iodo-substrates react more efficiently, while chloro-substrates exhibit slower conversion rates.

**Figure 4:**
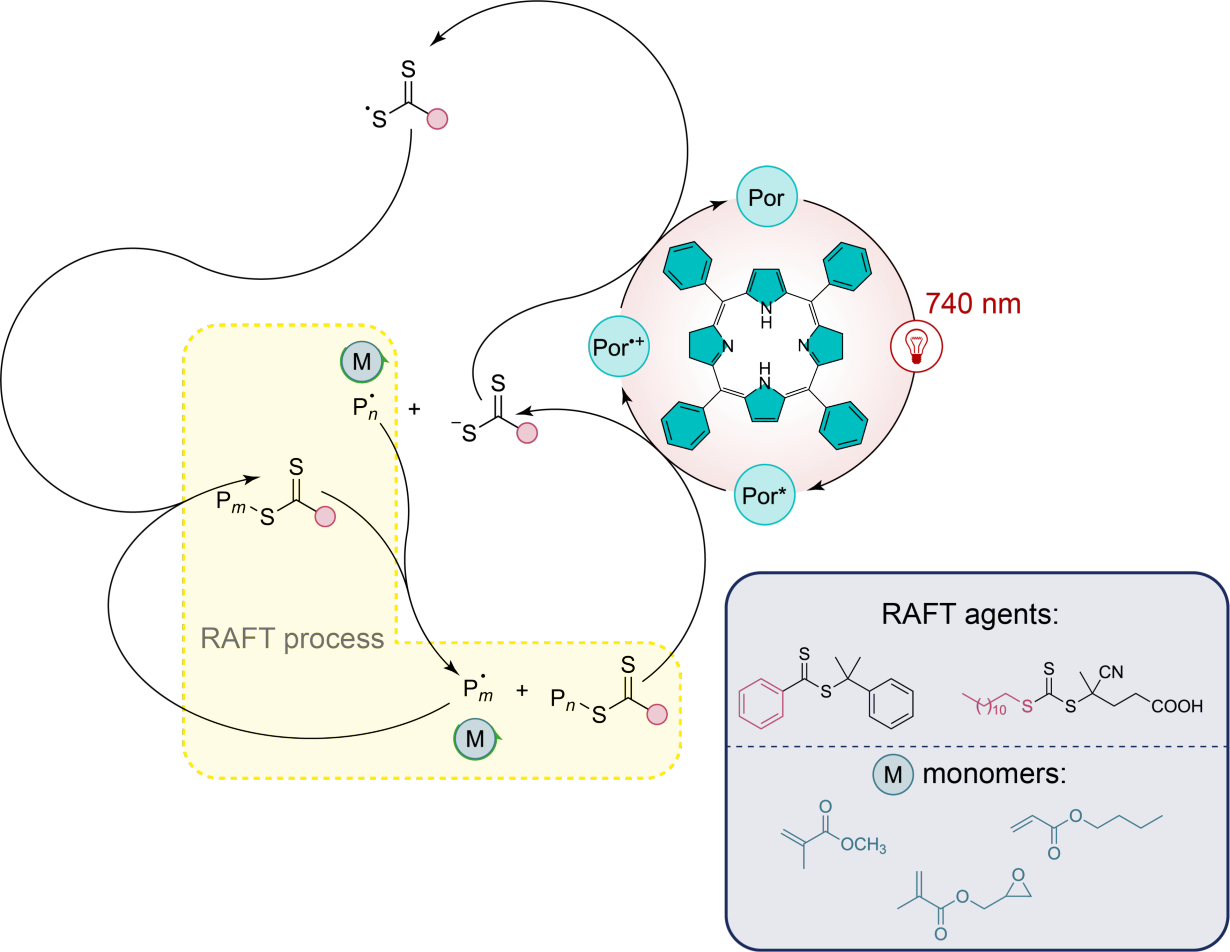
Photoinduced electron transfer-reversible addition-fragmentation chain transfer polymerization mediated by a red-light absorbing reduced tetraphenylporphyrin.

In a similar way, recent advances have shown that synthetic bacteriochlorins, inspired by natural photosynthetic pigments, can also serve as effective photocatalysts under even longer wavelengths. In particular, Zhang et al. have explored the application of a man-made bacteriochlorin for photoinduced electron transfer-reversible addition-fragmentation chain transfer (PET-RAFT) polymerization under far-red light (Figure 4) [43]. This synthetic bacteriochlorin, structurally modified from tetraphenylporphyrin with two reduced pyrrole rings, exhibits strong absorption in the far-red region, providing excellent control over molecular weight and polydispersity in the polymerization of various monomers, including methyl acrylate. The authors highlight that under red-light irradiation, the bacteriochlorin catalyst has achieved 89% monomer conversion in just 12 hours, with molecular weights closely matching theoretical predictions and low polydispersity indexes. Even with reduced catalyst loadings (down to 10 ppm), the polymerization process has remained efficient, maintaining high monomer conversions and well-controlled molecular weights. What makes this approach particularly innovative is its ability to operate in the presence of oxygen, overcoming a common limitation in radical polymerization, and its demonstrated efficacy in penetrating biological tissues up to 7 mm thick.

**Figure 5:**
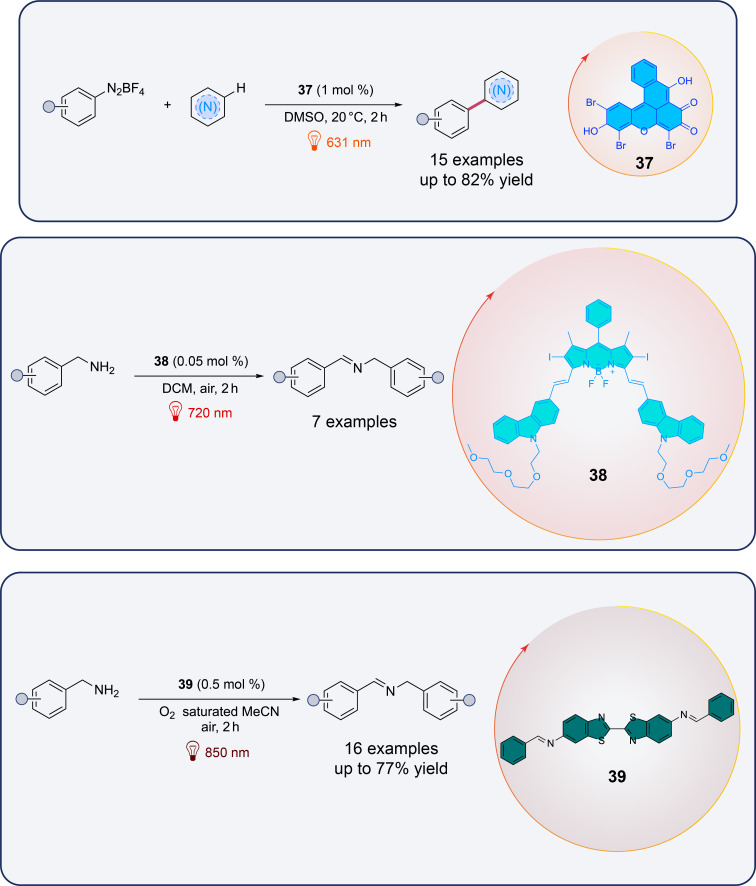
Recent examples of red-light-mediated photocatalytic reactions with traditional organic dyes.

Beyond the phthalocyanin/porphyrin family, other kinds of traditional organic photocatalysts have been extensively studied in the literature such as bridged Eosin Y **37** [44], BODIPY **38** [45], and dibenzothiazole **39** (Figure 5) [46]. Indeed, organic dyes, with their inherent advantages of low toxicity, environmental friendliness, and tunable optical properties, represent a promising alternative for red-light-mediated reactions. In particular, the versatility of these catalysts offers new avenues for facilitating red-light-driven chemical transformations with greater selectivity and efficiency.

**Figure 6:**
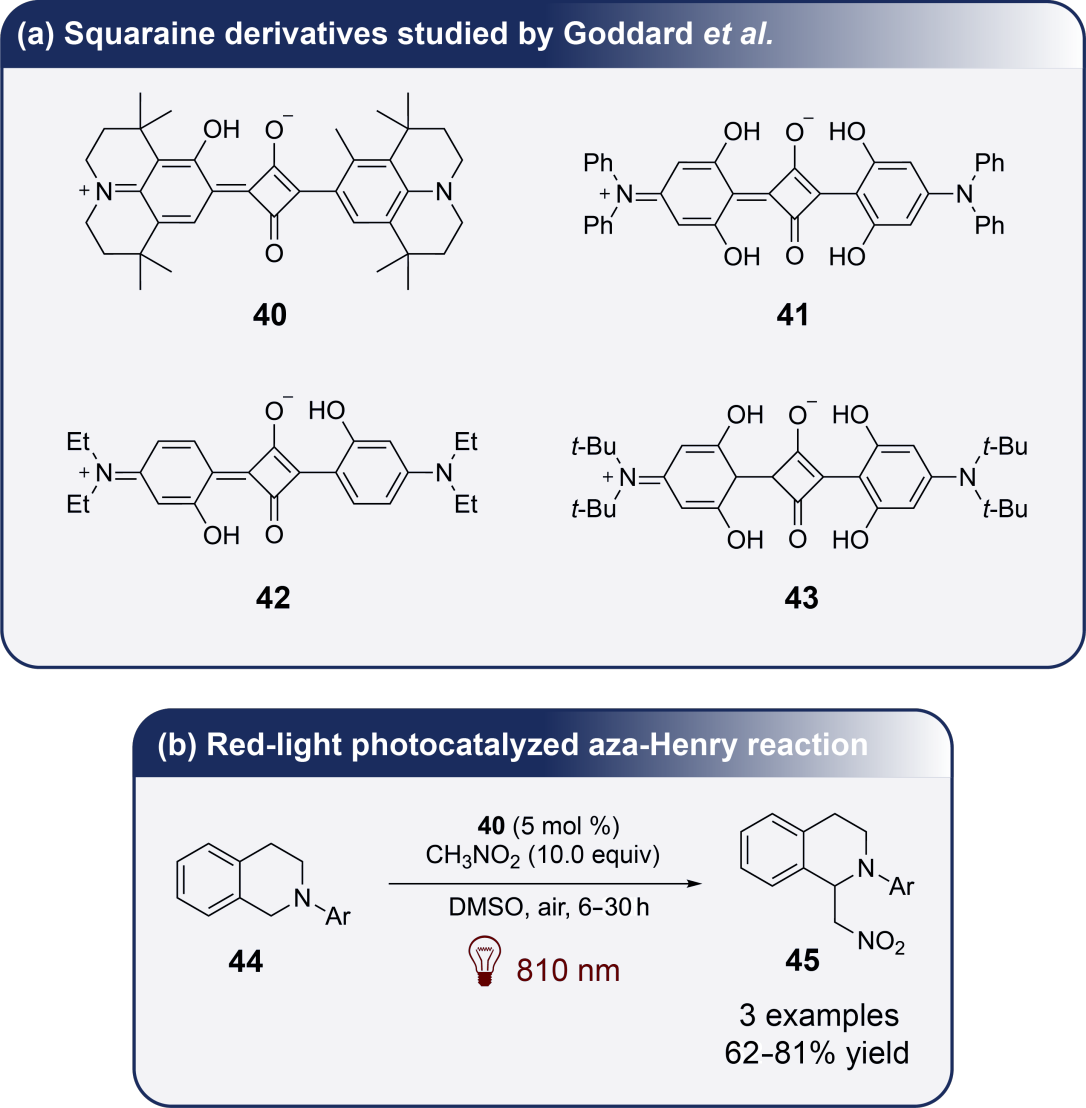
Squaraine photocatalysts used by Goddard et al. and aza-Henry reaction with squaraine-based photocatalyst **40**. Mechanism based on single-electron transfer and energy transfer.

Recently, the squaraine family, a class of organic compounds characterized by a four-membered unsaturated ring structure derived from squaric acid [47], has attracted attention due to its ability to promote single-electron transfer [48]. These compounds exhibit significant NIR fluorescence, making them valuable in applications such as biomolecule probing. Additionally, squaraine derivatives are well-established for their use in organic photovoltaic cells and as efficient photosensitizers for singlet oxygen generation. In 2022, Goddard et al. have reported the groundbreaking use of squaraine derivatives as novel organic NIR photocatalysts for various chemical transformations, marking the first application of these compounds in the field of photocatalysis [49]. The study has included a detailed mechanistic investigation to differentiate between competing single-electron transfer and energy transfer pathways. Through both experimental measurements and theoretical calculations, the authors have determined the redox potentials of several squaraine derivatives (**40**, **41**, **42**, and **43** presented in Figure 6a) which exhibit oxidation potentials at the excited state ranging from −1.22 V to −1.58 V vs SCE and reduction potentials at the excited-state between 0.84 V and 1.22 V vs SCE. In particular, derivative **40** has been proven to be the most efficient photocatalyst, promoting key transformations such as the aza-Henry reaction with **44** to give **45** under NIR light irradiation (Figure 6b). The reaction was found to critically depend on the presence of oxygen in the air for it to proceed. Through optimization and mechanistic investigations of the near-IR-photocatalyzed aza-Henry reaction, the authors have proposed either a single-electron transfer or an energy transfer mechanism. Additionally, the reaction displayed an unexpected sensitivity to the light wavelength used. Employing a higher-energy light source at 660 nm led to a reduction in the isolated yield from 70% to 56%. Despite the full conversion of the starting material, the overall efficiency was hindered by the formation of side-products. Furthermore, various nucleophiles were found to be compatible with this transformation.

**Figure 7:**
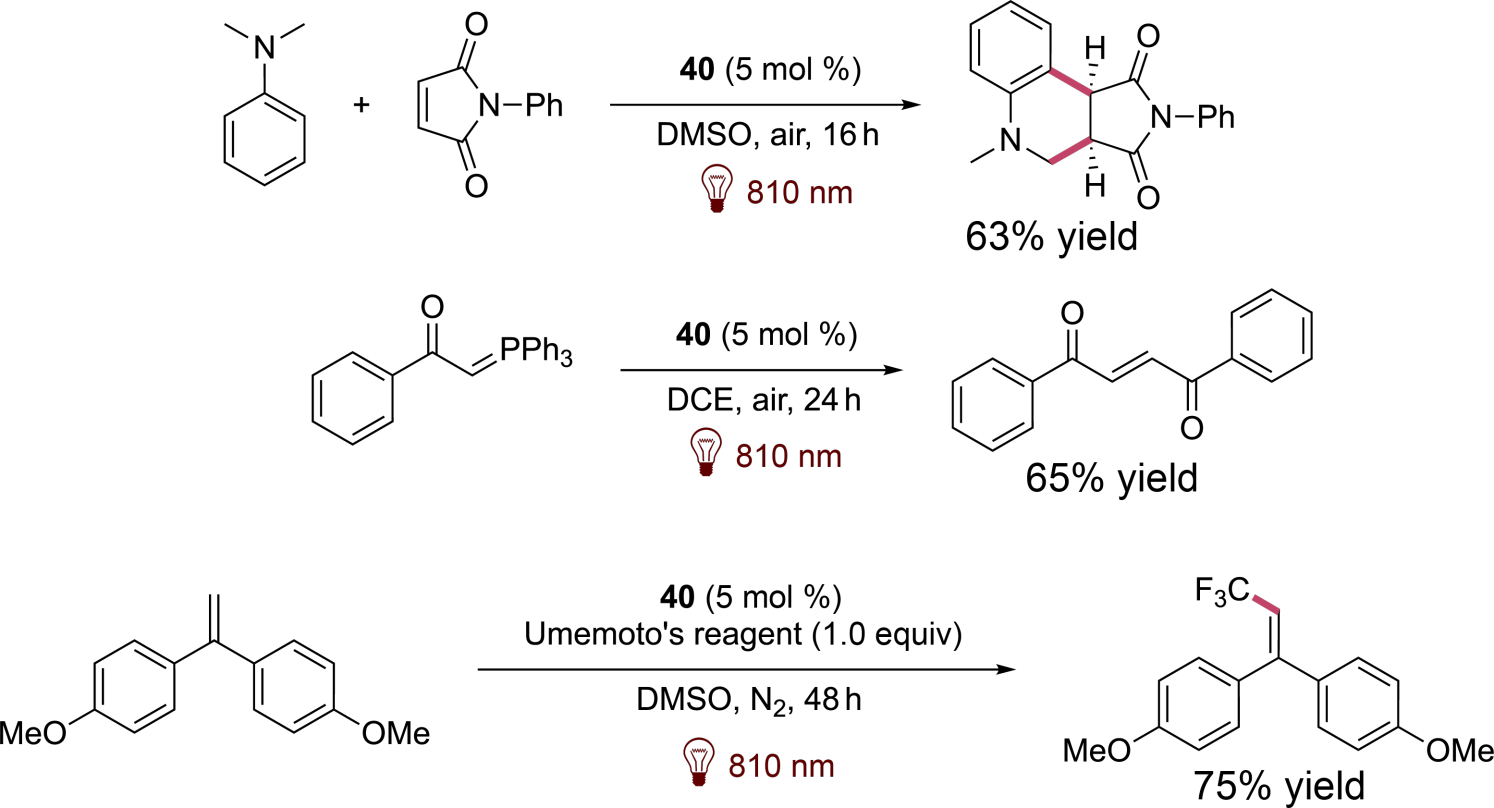
Reactions described by Goddard et al. involving **40** as the photocatalyst.

In parallel the same team further proposed various infrared-mediated reactions catalyzed by **40** as presented in Figure 7.

**Figure 8:**
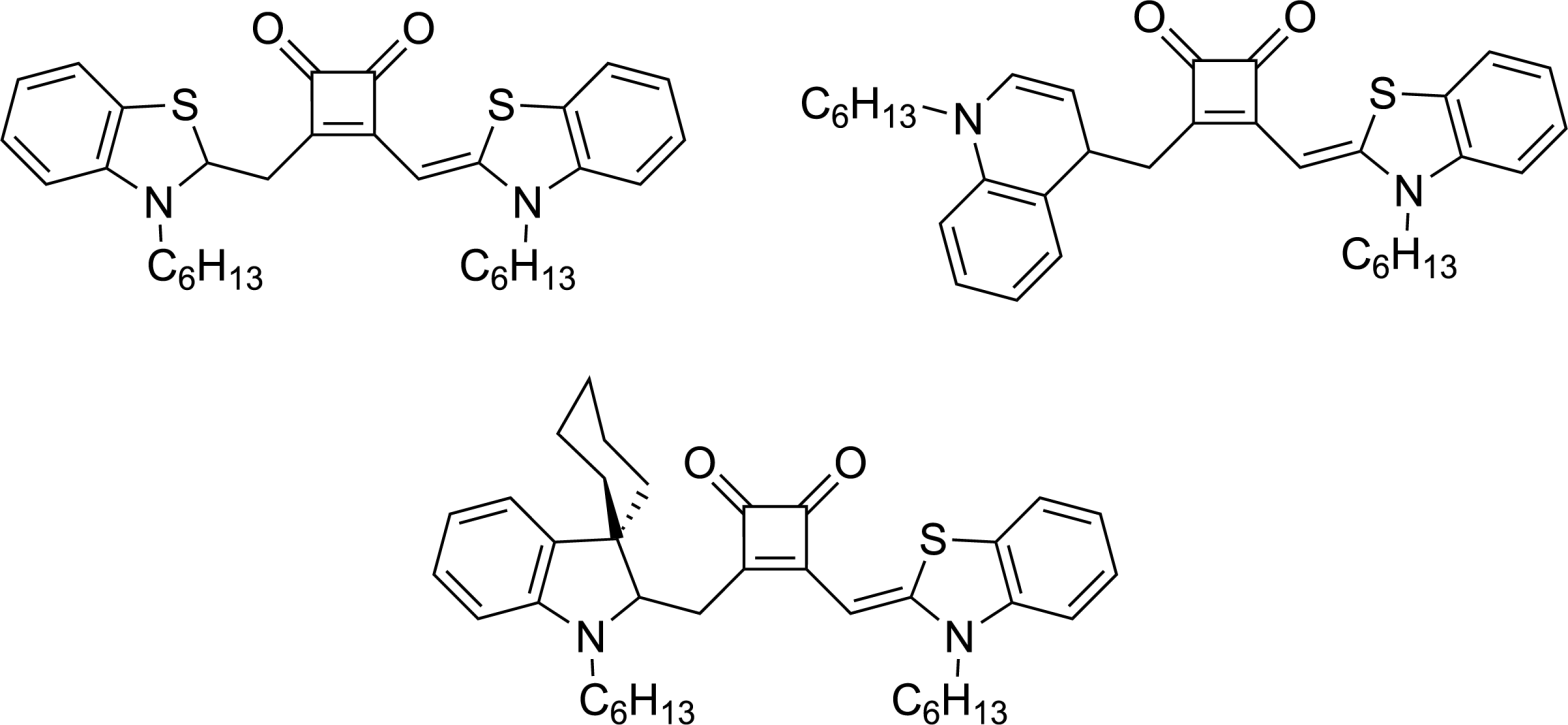
Various structures of squaraine derivatives used to initiate photopolymerizations.

It has to be noted that other squaraine derivatives have also been explored as innovative photoinitiators and photosensitizers for initiating the free radical polymerization (FRP) of methacrylates under near-infrared light exposure (Figure 8). Photopolymerization in this case is initiated by reduction of iodonium salts [50].

**Figure 9:**
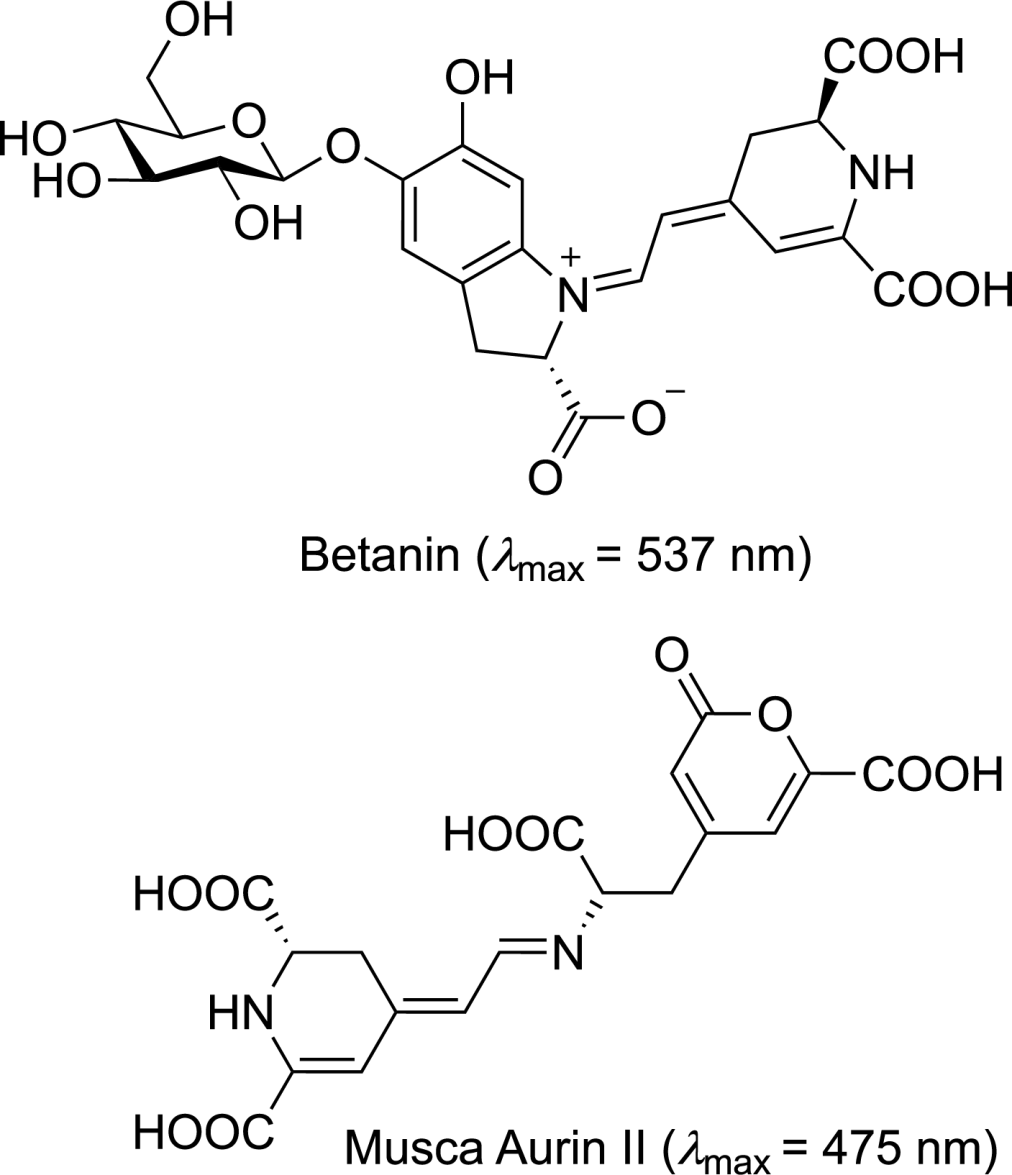
Naturally occurring cyanins.

Following the exploration of squaraine derivatives and their applications in NIR-mediated photocatalysis, another class of compounds, cyanin molecules, has recently gained significant attention for their unique photophysical characteristics and versatility in NIR-driven reactions [51]. Cyanins consist of nitrogen-containing heterocycles connected by a polymethin chain [52–53], whose synthesis and modifications have been widely explored [54–56]. Such compounds exhibit remarkable redox versatility, enabling them to participate in both oxidation and reduction reactions and few of them are naturally occurring such as Betanin and Musca-Aurin II (Figure 9). Their relatively stable excited states, combined with their low toxicity and ease of chemical modification, make them a highly effective platform for improving reaction efficiencies. These molecules can facilitate single-electron transfer or energy transfer under mild conditions, and their structural diversity allows for fine-tuning of their redox potentials and light absorption characteristics. This adaptability makes cyanins ideal for a variety of synthetic applications, such as polymerization and radical-mediated reactions, often difficult for catalysts operating under visible light.

**Figure 10:**
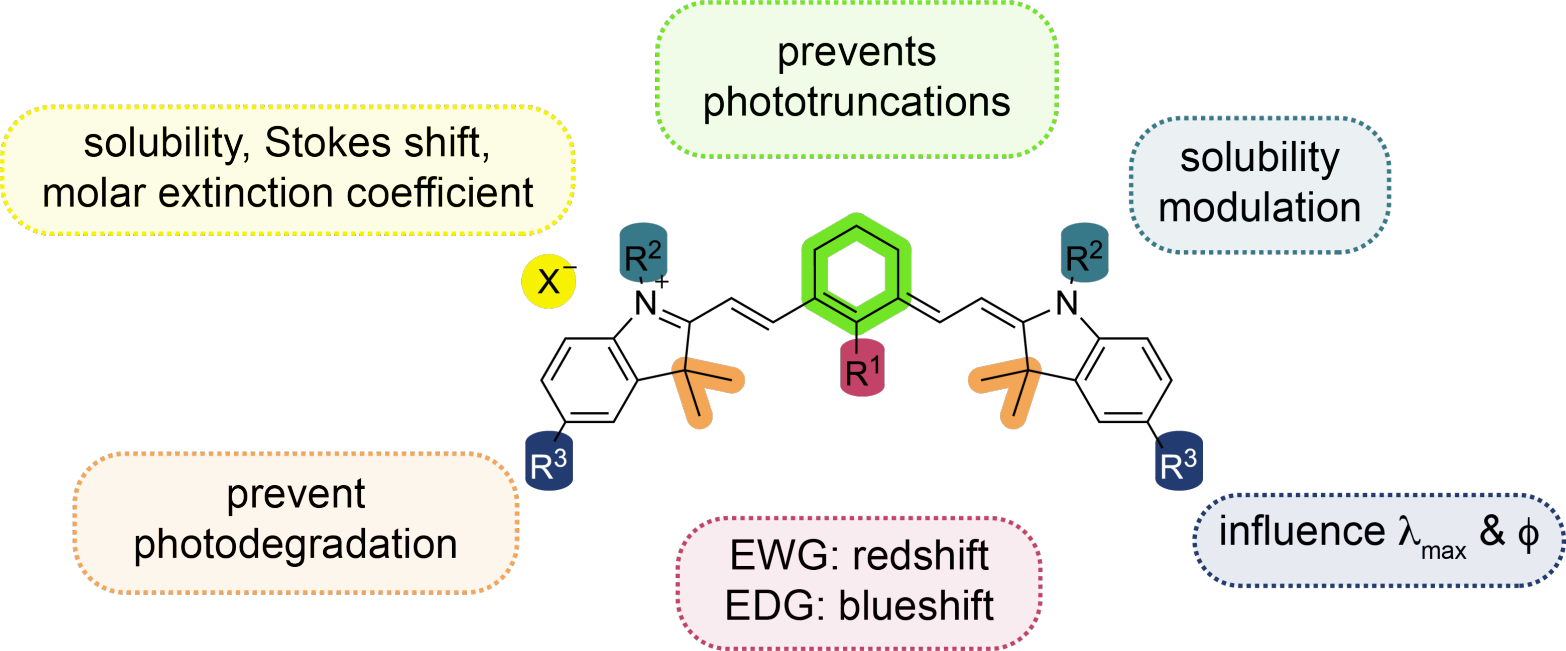
Influence of the structure on the photophysical properties of a cyanin dye.

Cyanin compounds can be categorized based on their structural motifs, such as closed-chain cyanins, streptocyanins, and merocyanins. These groups are distinguished by their terminal structures, which can range from heterocyclic to non-heterocyclic rings or even amino and carbonyl groups. Furthermore, cyanins can also be classified by the number of methine units in their polymethine chains, including monomethin, trimethin, pentamethin, and heptamethin derivatives. The length of the polymethin chain has a pronounced effect on their absorption properties, with the addition of each conjugated carbon–carbon double bond causing a red shift of about 100 nm in the absorption spectrum. The formation of *J*-aggregates allows cyanin compounds to narrow both absorption and emission peaks, along with a bathochromic shift in their spectra. Heptamethin cyanins, in particular, are highly relevant for NIR applications, making them valuable in photochemistry. Additionally, their solubilities, redox and photophysical properties can be fine-tuned through specific chemical modifications, further enhancing their versatility for a range of synthetic and photophysical applications as well as preventing their photodegradation (Figure 10) [57–60].

**Figure 11:**
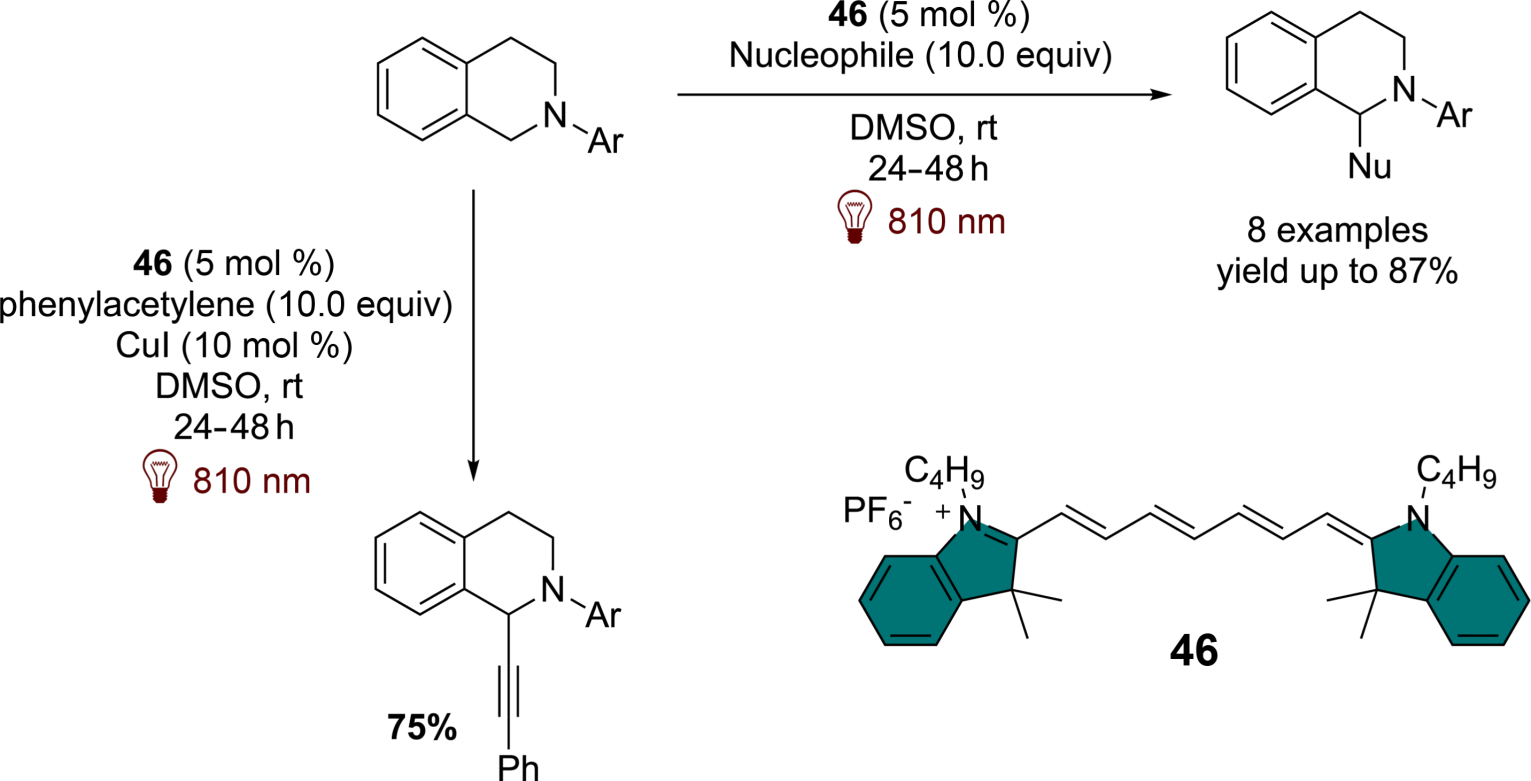
NIR-light-mediated aza-Henry reaction photocatalyzed by **46**.

The use of cyanins as near-infrared photosensitizers was first introduced in photopolymerization processes. Cyanins have been shown to effectively initiate radical polymerization under visible light through mechanisms such as borate oxidation [61] and 1,3,5-triazine reduction [62]. More recently, cyanin dyes have enabled the reduction of iodonium salts under NIR excitation [63–64]. These preliminary findings suggest that cyanins, specifically tailored to absorb in the NIR region, exhibit promising redox properties for applications in organic synthesis. A recent work by Goddard et al. has demonstrated that compound **46** is highly effective in various photoredox transformations, such as aza-Henry reactions with nucleophiles like malonates, cyanides, and phosphites. The study further revealed that the radicals generated from these processes can be successfully utilized in dual catalysis with copper, yielding a variety of alkynylated products (Figure 11) [65].

**Scheme 13:**
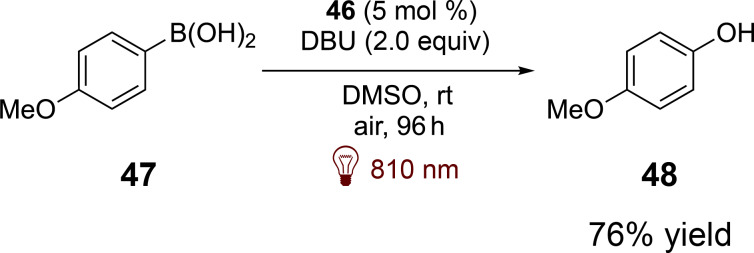
Photocatalyzed arylboronic acids oxidation by **46**.

Compound **46** has been shown to exhibit redox properties similar to those of squaraine derivatives, enabling key transformations such as the cyclization of anilines with maleimides and the reduction of Umemoto salts for trifluoromethylation of alkenes. With an excited-state reduction potential around 0.80 V, **46** demonstrates the capability to oxidize boronic acids such as **47**, producing the corresponding alcohols **48** via a single-electron-transfer mechanism that leverages atmospheric oxygen (Scheme 13).

**Figure 12:**
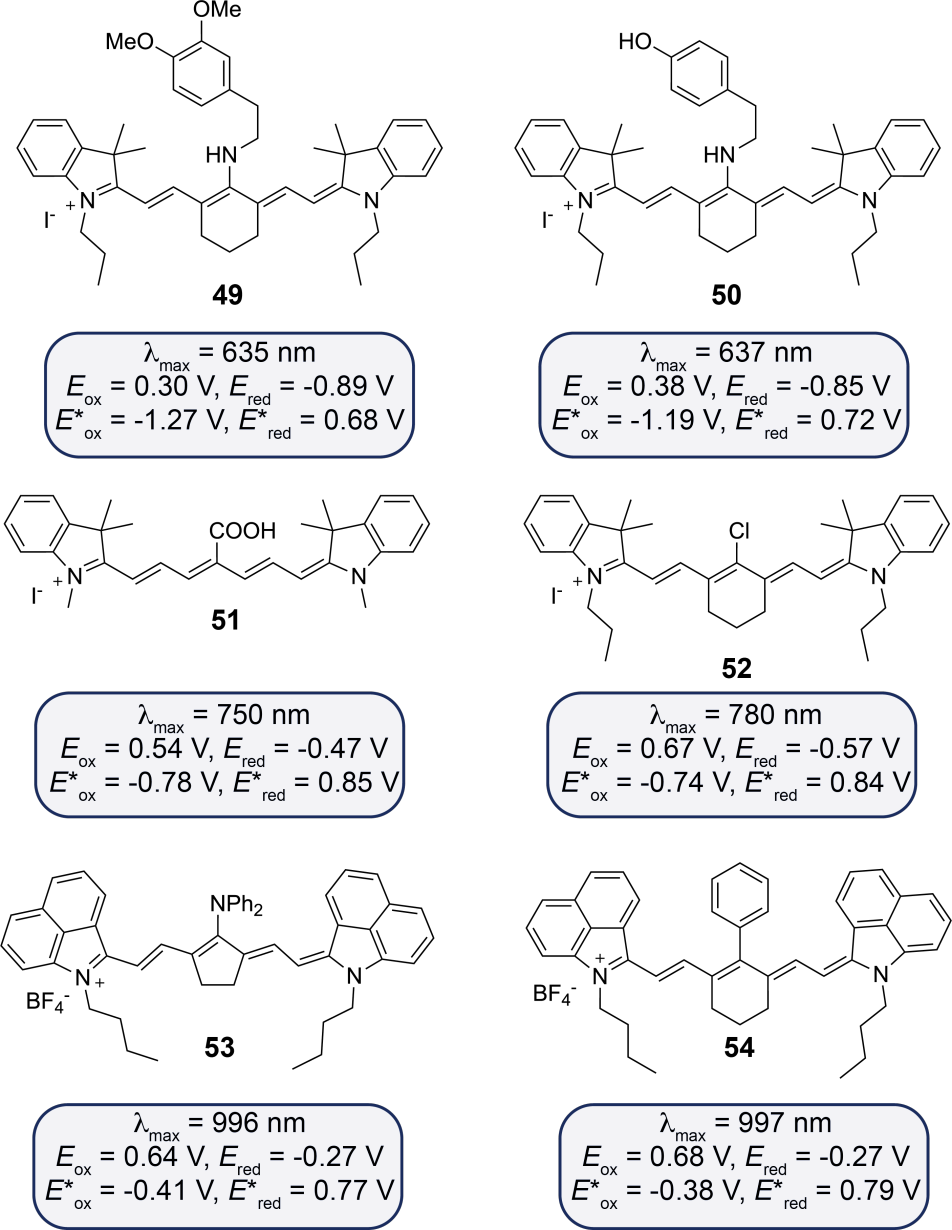
Cyanin structures synthetized and characterized by Goddard et al. (redox potentials given against saturated-calomel electrode (SCE)).

While the initial photocatalytic results using NIR-irradiated **46** were promising, the overall reaction kinetics were relatively slow. In response, Goddard et al. have developed a second generation of cyanin dyes. For example, in the case of the photocatalyzed trifluoromethylation of alkenes, it has been shown that the stability of the photocatalyst was crucial for achieving efficient and faster conversion. In their study, the authors have reported the synthesis and characterization of over 20 new cyanins, reporting their synthetic applications and both calculated and measured their photophysical properties and redox potentials [66]. The study shows that substituents at the central position of the polymethin chain significantly affect their redox properties (Figure 12). Cyanins with electron-donating groups (e.g., **49** and **50**) are less oxidizing, with cathodically shifted reduction potentials compared to the unsubstituted **46**. In contrast, cyanins with electron-withdrawing groups (e.g., **51** and **52**) show anodically shifted reduction peaks, making them more oxidizing. Extended conjugation in cyanins (e.g., **53** and **54**) further increases their oxidizing nature at the ground state. Overall, both central substitution and extended conjugation play crucial roles in determining the redox behavior of these dyes. Cyanins with an amino group on the heptamethin chain have been proven to be the most effective photocatalysts for accelerating the aza-Henry reaction. The differing reaction kinetics and tests with 9,10-dimethylanthracene suggest that a cooperative mechanism involving both single-electron transfer and singlet oxygen generation via energy transfer likely drives the product formation. In a related study on the reduction of Umemoto salts for trifluoromethylation of alkenes, most photocatalysts showed instability under the experimental conditions. However, **54** has emerged as the most stable and efficient NIR photocatalyst, facilitating the trifluoromethylation even under low-energy irradiation at 940 nm.

**Figure 13:**
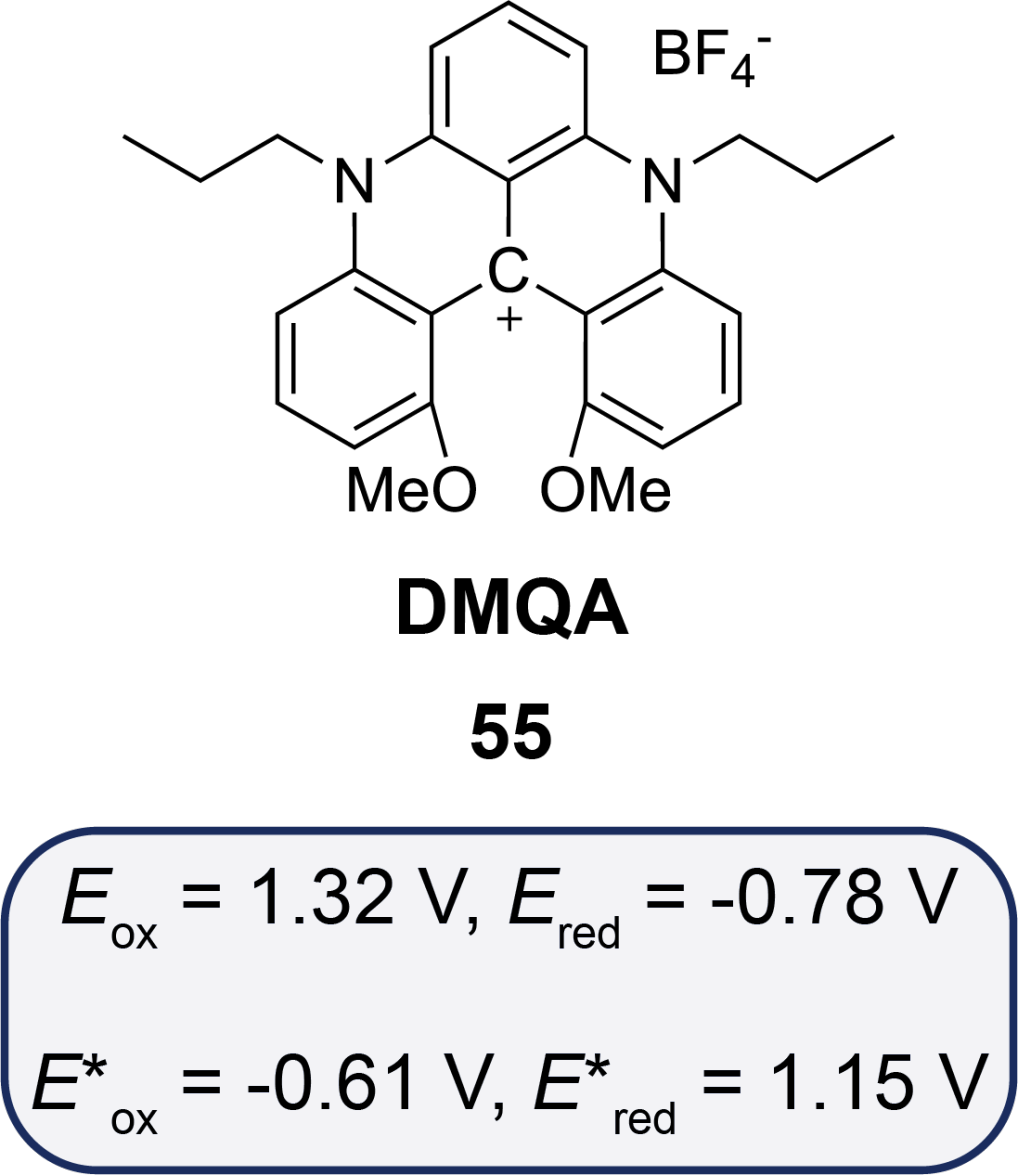
*N*,*N*′-Di-*n*-propyl-1,13-dimethoxyquinacridinium (**55**) with its redox potentials at its ground state and excited state (values given against saturated-calomel electrode (SCE)).

Another emerging class of catalysts that has garnered attention for its applications in NIR photocatalysis is helical carbenium ion-based systems. These systems, characterized by their distinct helical structures and robust redox behavior, represent a novel approach to harnessing NIR light for challenging chemical transformations. The helical carbenium ion, dimethoxyquinacridinium (DMQA^+^) and its synthesis, has been extensively studied for its photophysics, making it a subject of significant interest in the field of photocatalysis [67–68]. Based on previously observed results, Gianetti et al. have speculated that *N*,*N*′-di-*n*-propyl-1,13-dimethoxyquinacridinium (**55**, DMQA) tetrafluoroborate could serve as a versatile NIR organic photocatalyst [69]. Redox potentials of this photocatalyst engaged in both oxidative quenching and reductive quenching cycles have been calculated and have indicated that this latter could be a viable candidate for use in photoredox catalysis. Additionally, its measured excited-state lifetime (τ = 5.5 ns) is similar to those of other widely used organic photocatalysts, such as carbazole derivatives, further supporting its potential applicability [70]. Values are given in Figure 13.

**Scheme 14:**
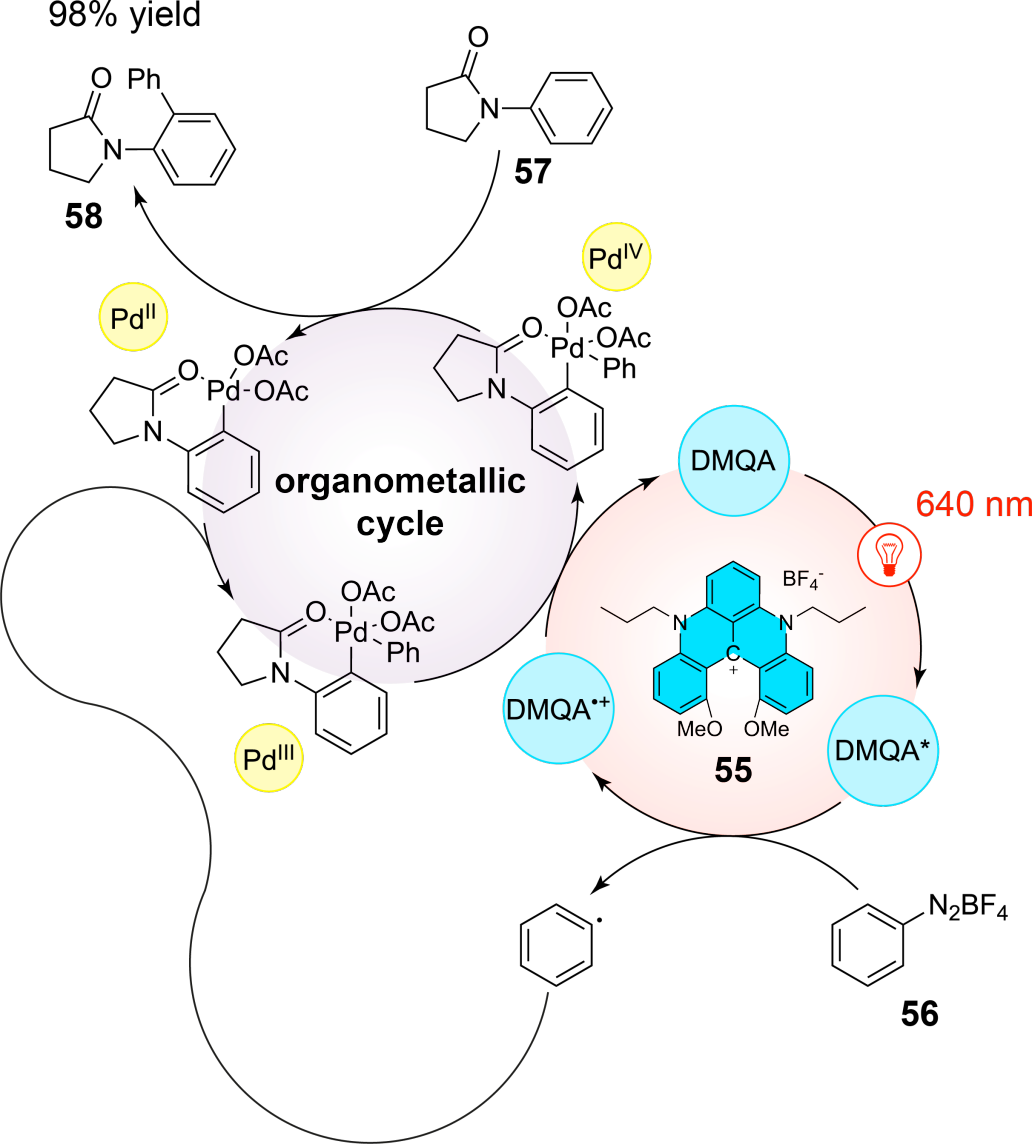
Dual catalyzed C(sp^2^)–H arylation of **57** using DMQA **55** as the red-light-absorbing photocatalyst.

The first results obtained in organic synthesis have consisted in the dual Pd/DMQA-catalyzed C(sp^2^)–H arylation with aryldiazonium such as **56** with lactam derivative **57**, which have leaded to similar results as the traditionnal use of Ru(bpy)_3_^2+^ with the formation of **58** in 95% yield (Scheme 14).

**Scheme 15:**
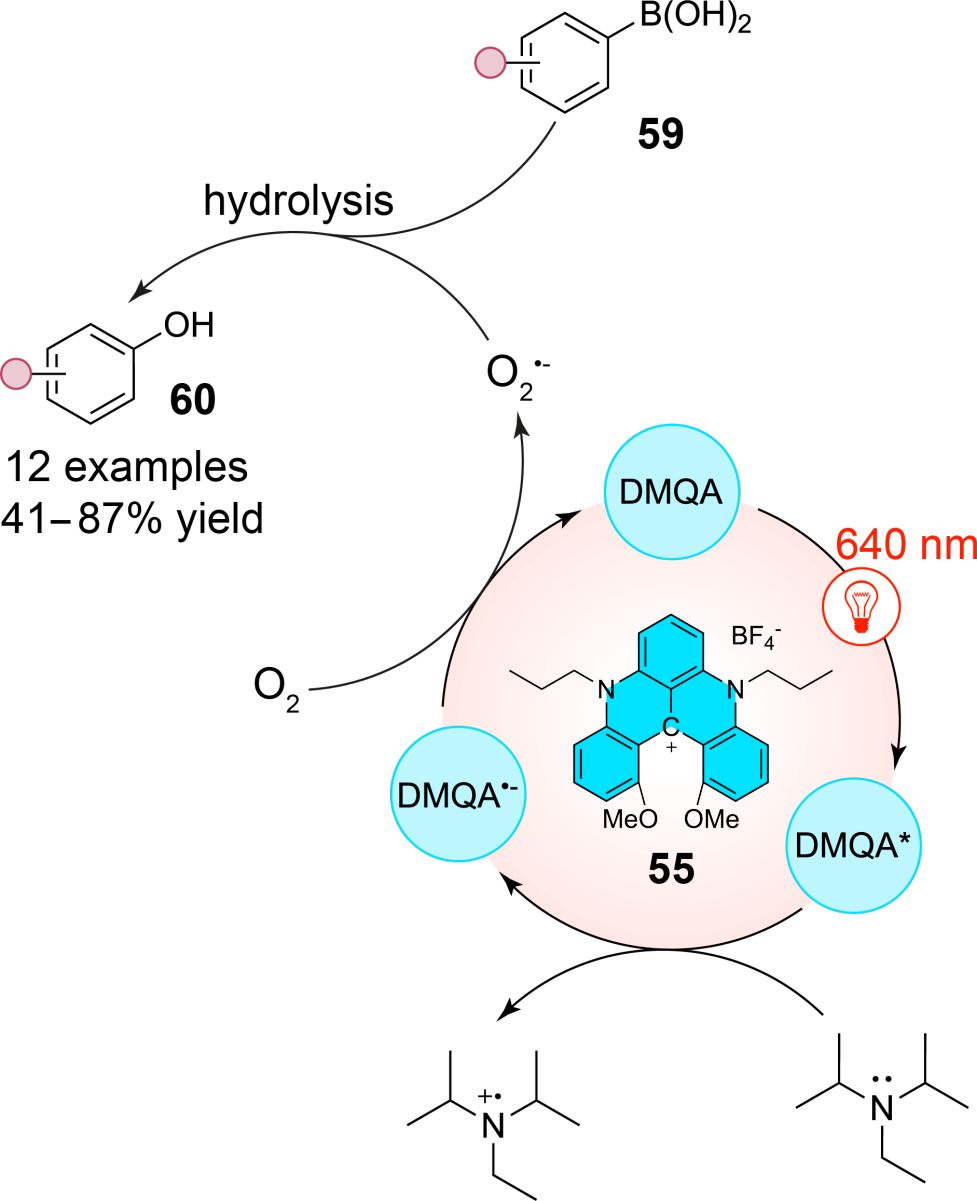
Red-light-mediated aerobic oxidation of arylboronic acids **59** into phenols **60** via the use of DMQA as the photocatalyst.

Similarly, the photoinduced aerobic oxidative hydroxylation of arylboronic acids **59** has been successfully accomplished. Since this reaction does not depend on the presence of singlet oxygen, it confirmed the electron-transfer capability of the DMQA catalyst, which operates through a reductive quenching mechanism. A suitable reaction pathway was established, leading to moderate to excellent yields of the corresponding phenols **60** (Scheme 15). The reaction primarily involves the oxidation of iPr_2_NEt (DIPEA) by the excited photocatalyst **55** to generate the radical cation iPr^2^NEt^•+^ (iPr_2_NEt/iPr^2^NEt^•+^ = +0.72 V vs SCE) and the reduction of O_2_ by the reduced photocatalyst, forming the superoxide radical anion O_2_^•−^ (O_2_/O_2_^•−^ = −0.57 V vs SCE). This latter can then react with arylboronic acids **59** to give, after hydrolysis, phenol derivatives **60**.

**Figure 14:**
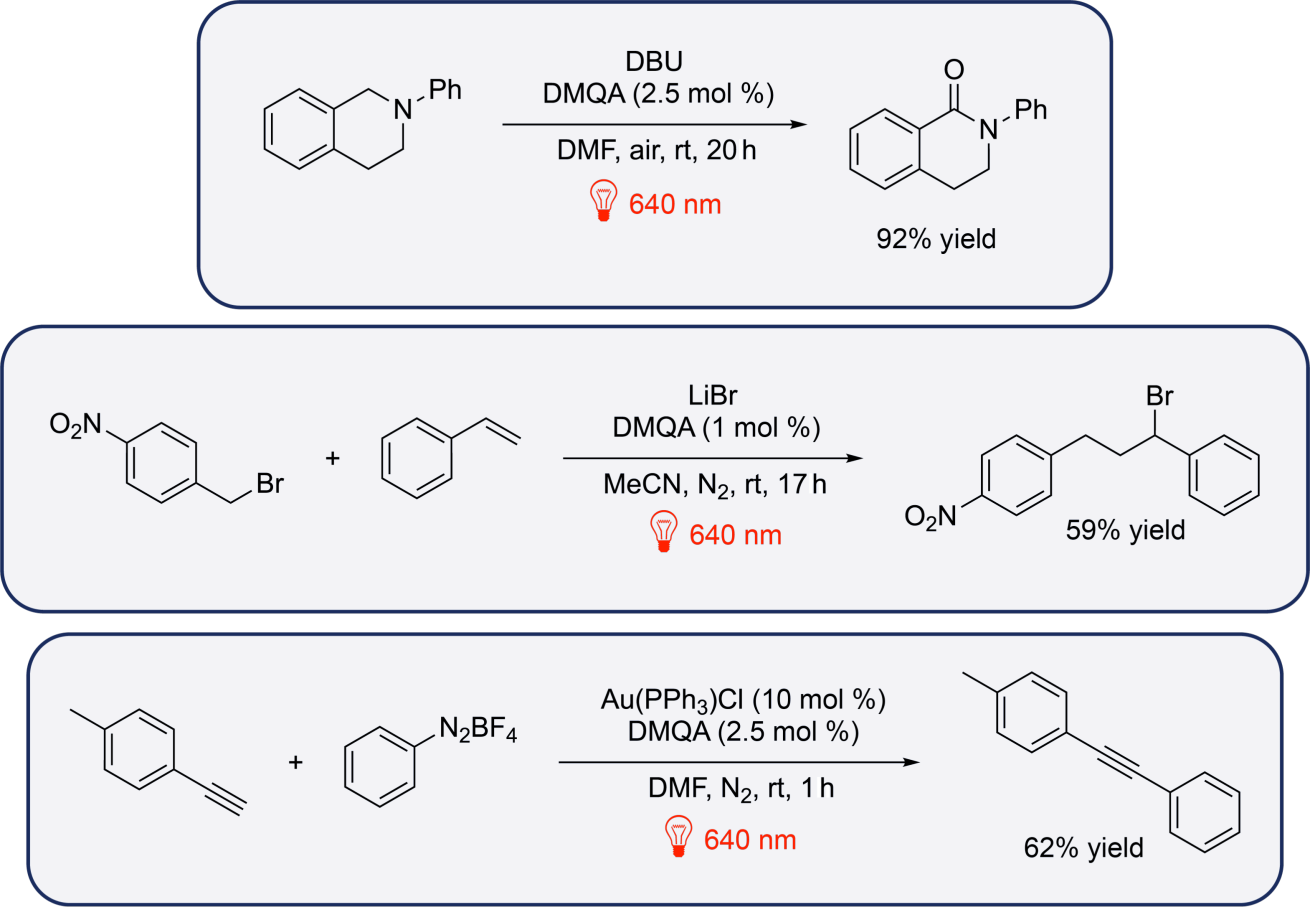
Red-light-photocatalyzed reactions proposed by Gianetti et al. using DMQA as the photocatalyst.

Other transformations have been addressed by Gianetti et al. such as C(sp^3^)–H oxidation, intermolecular atom transfer radical addition and C(sp)–H arylation using red light and DMQA (Figure 14), hence showing the great versatility of this photocatalyst.

### Red-light photocatalysis in biological systems

Photochemical reactions are of high interest for being applied in biological or medicinal domains. However, the weak light penetration of biological tissues limits the application to surface processes. The penetration of NIR light is considerably better [71]. Consequently, many photochemical processes are currently studied using NIR light [72]. This approach considerably enlarges the application of photochemical reactions to medicine.

**Scheme 16:**
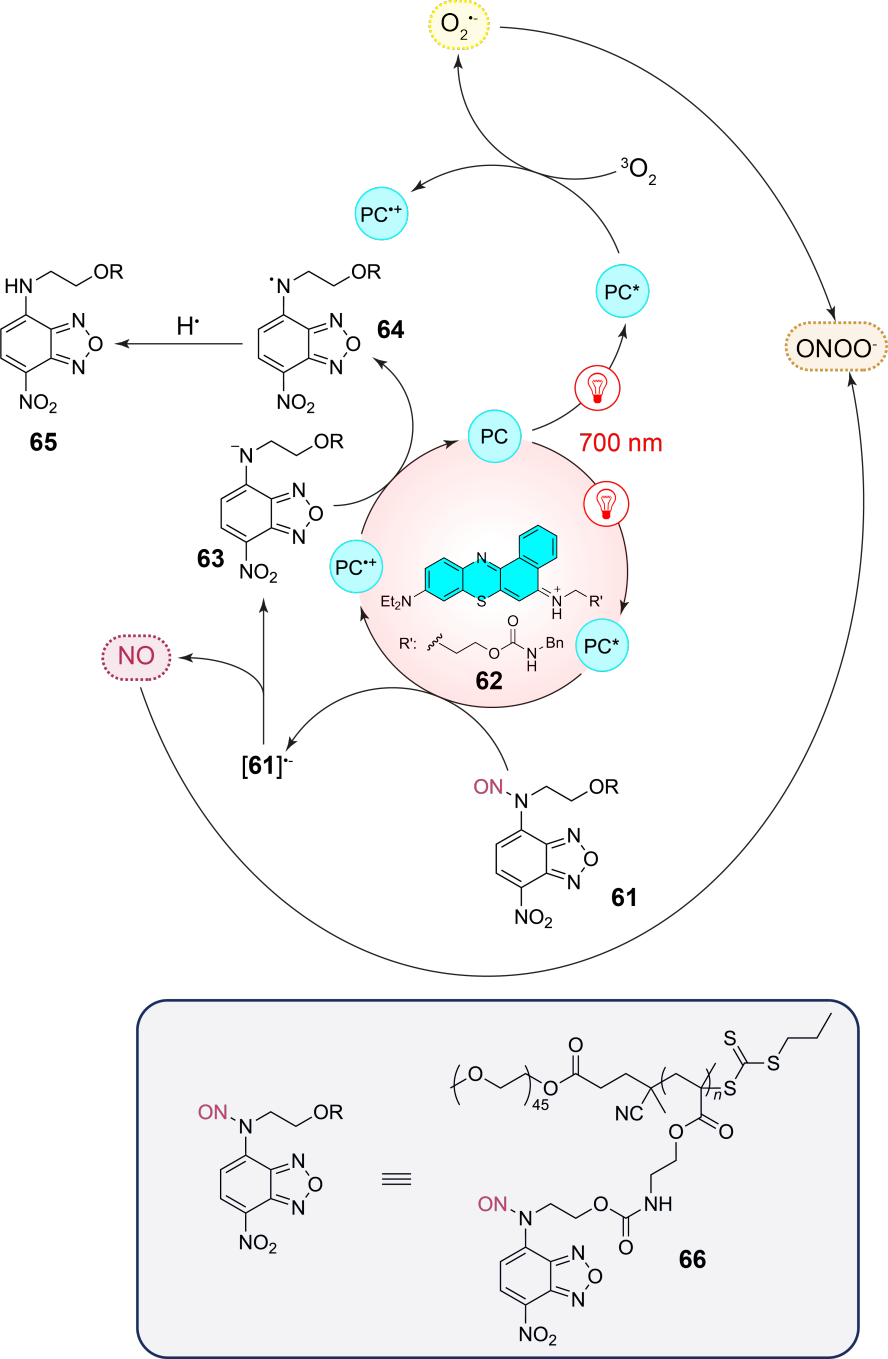
Simultaneous release of NO and production of superoxide (O_2_^•−^) and their combination yielding the peroxynitrite anion (ONOO^−^).

The peroxynitrite anion (ONOO^−^) plays an important role in many diseases such as diabetes, neurodegenerative disorders, or inflammatory diseases [73]. In order to study the mechanisms of the biological activity and to find biomedical applications, different releasing systems of this anion are developed. The peroxynitrite anion is formed by coupling of nitric oxide (NO) and the superoxide (O_2_^•−^). An efficient system has been developed to realize this reaction (Scheme 16) [74]. In a photocatalytic reaction, NO is released from the corresponding compound **61**. Nile blue **62** is used as photocatalyst (**PC**). After photochemical excitation with NIR light (λ = 700 nm), electron transfer occurs from the catalyst to compound **61**. This step is most probably favored by the formation of a precursor complex involving π–π-stacking [75]. The resulting radical anion releases NO also yielding the anion **63**. Electron transfer to the radical cation of the photocatalyst regenerates it. In this step, the neutral radical **64** is also formed. Hydrogen abstraction (hydrogen atom transfer, HAT) yields compound **65**. NO and the superoxide (O_2_^•−^) are simultaneously produced by the same photocatalytic system. In order to favor their combination leading to the peroxynitrite anion (ONOO^−^), the NO-releasing structure is incorporated in a polymeric structure **66** which generates micellar nanoparticles. The substituent R’ in the photocatalyst **62** favors its incorporation in the nanoparticles. Thus, both reaction partners are approached.

**Figure 15:**
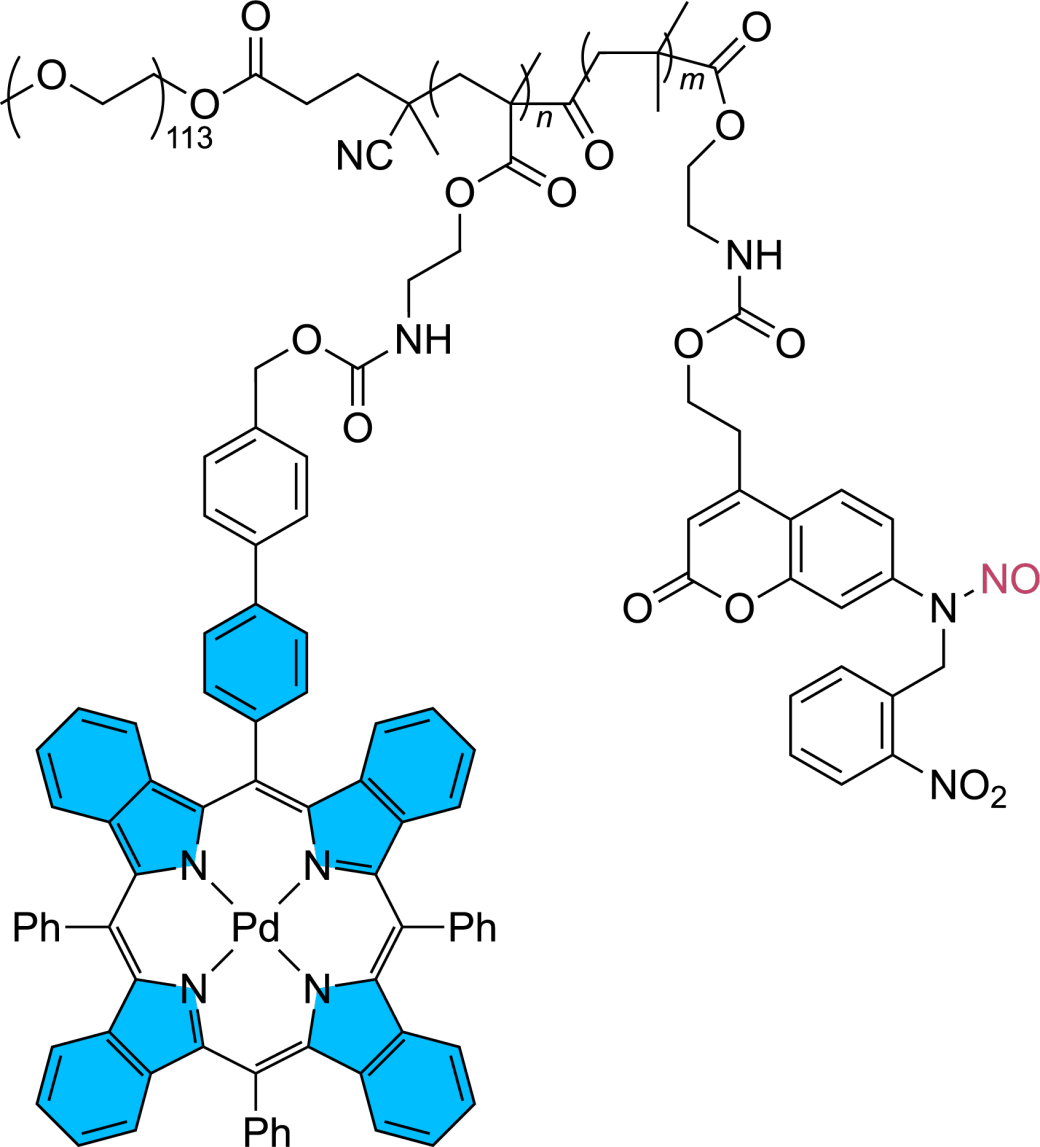
Palladium porphyrin complex as the photoredox catalyst and the NO releasing substrate are linked in the same block copolymer.

A similar photocatalytic system has been developed for the release of nitric oxide (Figure 15) [76]. Meanwhile, numerous applications of the NO release to medicine have been studied [77]. In the present case a palladium porphyrin complex is used as photocatalyst and a coumarin derivative is used as NO donor when the irradiation is carried out at λ = 630 nm. NO is released in a photoredox catalytic process and both reaction partners are covalently bound in a block copolymer which forms micelles. This method has been developed in the context of the treatment of intervertebral disc degeneration caused by bacterial infection. It also inhibits the inflammatory response and osteoclast differentiation in the intervertebral disc tissues. The present coumarin derivative is also capable of releasing nitric oxide by direct UV light absorption. However, this method, although simpler, is not suitable for the present medicinal application.

**Scheme 17:**
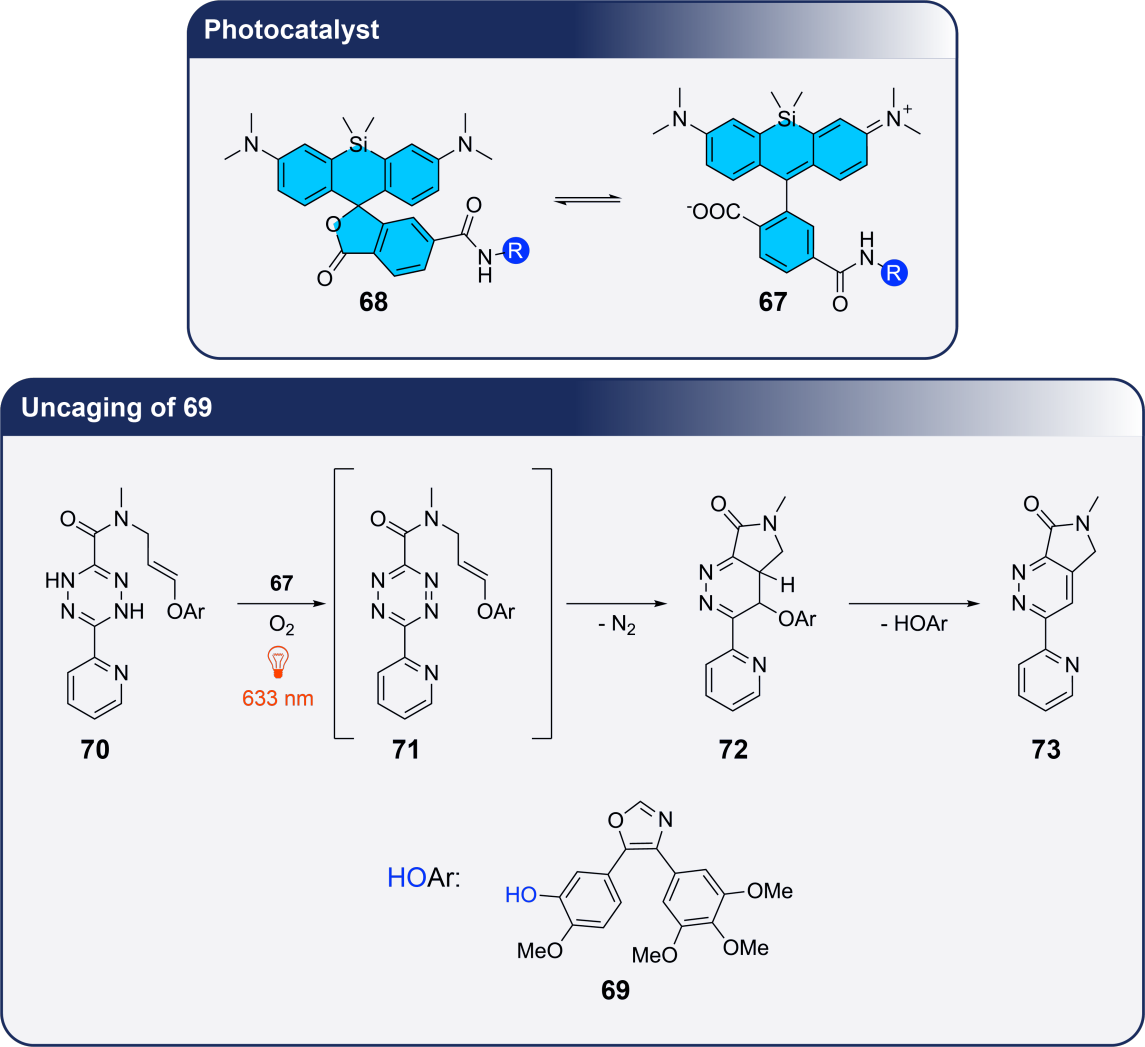
Uncaging of compound **69** which is a microtubule depolymerizing agent using near IR irradiation. The sensitizer is attached to tubulin with the docetaxel conjugate (R).

The space and time-controlled release of bioactive compounds is an important tool in bio- and medicinal chemistry to study biochemical mechanisms or to develop new therapeutic methods [78–80]. Such reactions can be performed at particular locations such as DNA or tubulin, when the photocatalyst is placed via a tethered ligand (Scheme 17) [81]. In the present case, the triarylmethine dye **67** was used as sensitizer. It is in equilibrium with the lactone form **68** and can bind either to tubulin or DNA depending on the conjugate R. The lactone form enables cell-permeability. The bioactive compound **69** causes microtubule depolymerization and it is caged in the dihydrotetrazine derivative **70**. Upon photocatalyzed oxidation, the corresponding tetrazine compound **71** is formed. This reaction only occurs close to the photocatalyst or sensitizer fixed at the intracellular target, for example at tubulin. An intramolecular Diels–Alder reaction followed by nitrogen extrusion generates the intermediate **72**. In the following uncaging step, the active compound **69** is released and aromaticity is regenerated in the pyrazine derivative **73**. An extracellular reaction of the system is quenched by ascorbate (low permeability) thus inhibiting the photooxidation of **70**.

**Scheme 18:**
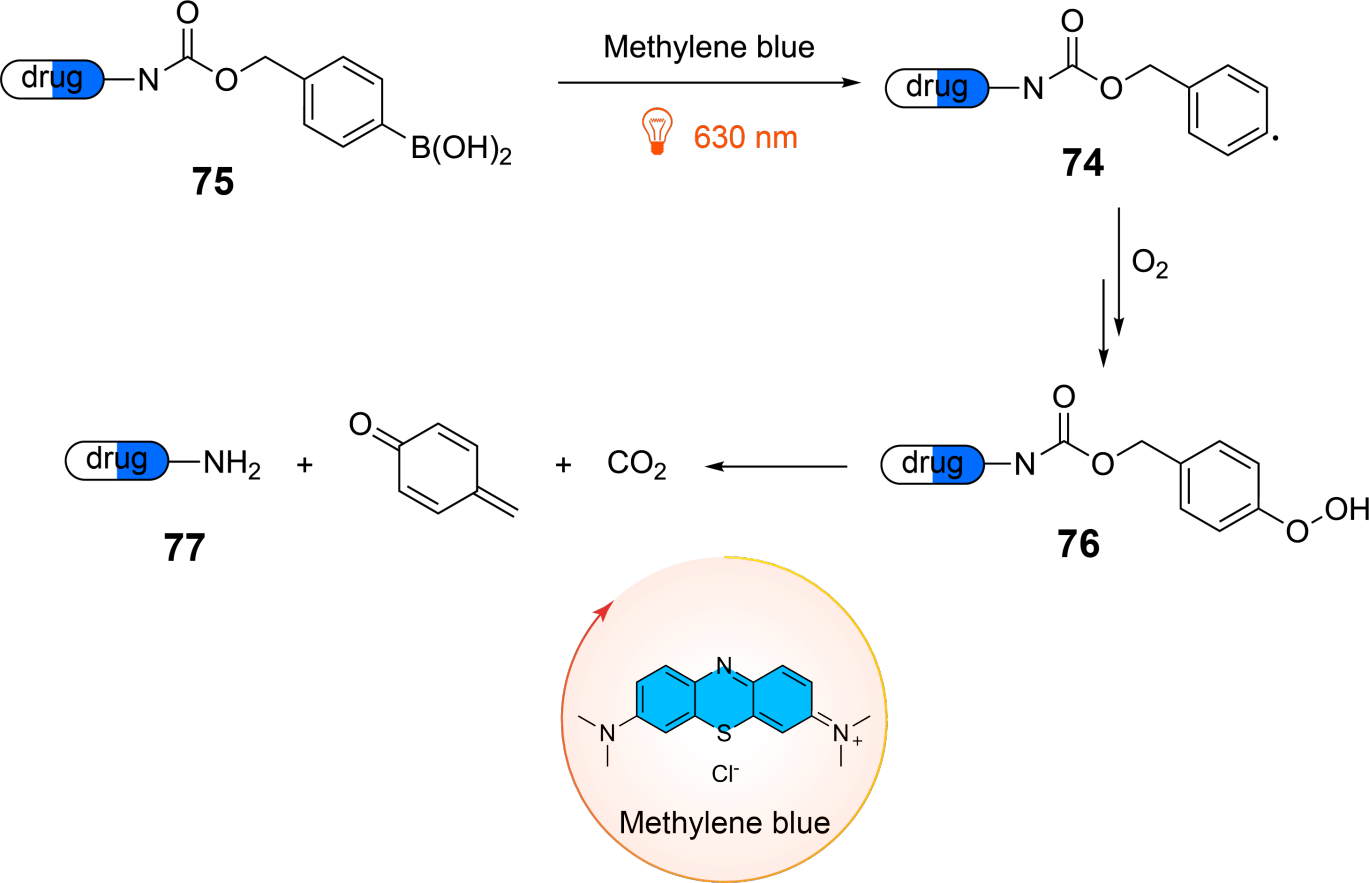
Photochemical uncaging of drugs protected with a phenylboronic acid derivative using near IR irradiation.

Systematic uncaging of drugs is an important topic in medicinal chemistry and general methods for this purpose are of high interest. Phenyl radicals **74** can be produced from the corresponding arylboronic acid precursors **75** (Scheme 18) and the addition of oxygen leads to the hydroperoxide **76** [82]. The latter undergoes fragmentation releasing the drug, quinomethene **77**, and carbon dioxide. By this route baclofen, vorinostat, or AA147 were uncaged.

**Scheme 19:**
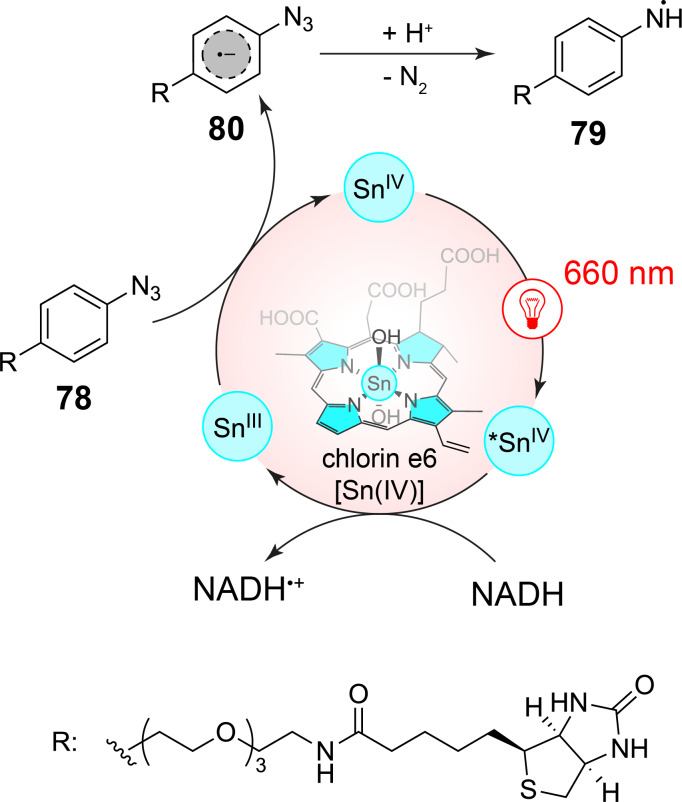
Photoredox catalytical generation of aminyl radicals with near IR irradiation for the transfer of biotin to biological structures.

Near IR irradiation (λ = 660 nm) can also be used to transform aromatic azides **78** into corresponding aminyl radicals **79** in a photoredox catalytic process (Scheme 19) [83]. In this case, a tin chlorin e6 complex [Sn(IV)] is used as photoredox catalyst, which, after photochemical excitation, is easily reduced to [Sn(III)]. Among the investigated compounds for this purpose, NADH was the best reductant. The tin(III) species reduces the azide **78** and the resulting radical anion **80** yields aminyl radicals **79** after protonation and release of nitrogen. These radicals have been added to enzymes such as carbonic anhydrase which in this way was labeled in vitro with biotin. In a cellular context, the tin chlorin e6 complex [Sn(IV)] was also conjugated to antibodies and thus transferred to epidermal growth receptor (EGFR), a cell surface receptor tyrosine kinase. Under these conditions, biotinylation was carried out only at the cell surface close to the sensitizer marked antibodies. The technique was also applied to the labeling at the erythrocyte surface. The method was studied as well with more conventional iridium-based photoredox catalysts absorbing light in the range of 390 < λ < 470 nm. However, tissue penetration of light of these wavelengths is only 1–2 mm while the tissue penetration of light of λ = 660 nm is > 6 mm. Consequently, these conditions were significantly less efficient. A similar study has been published using osmium-based photosensitizers also absorbing light in the near infrared domain [84].

**Scheme 20:**
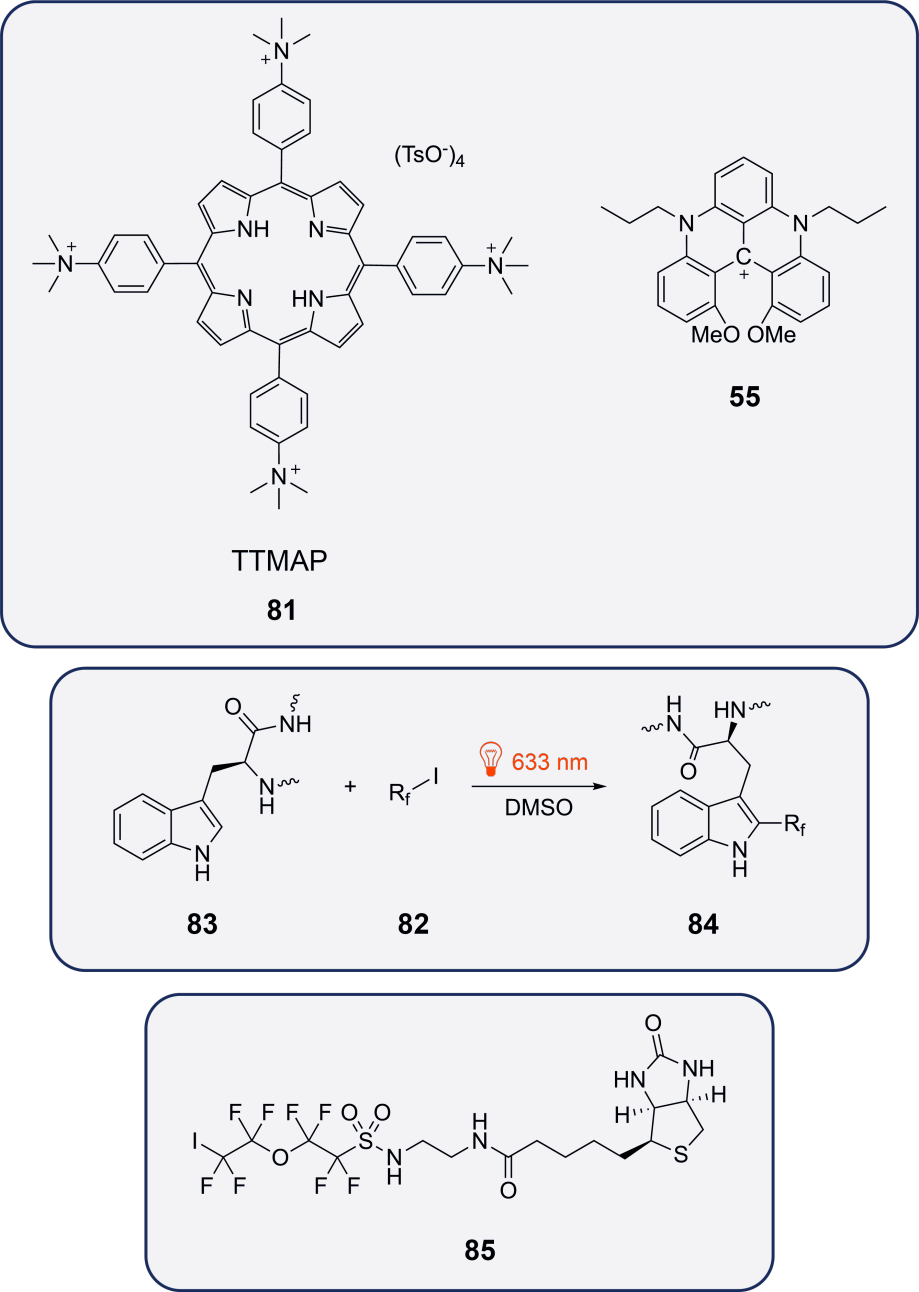
Photoredox catalytical fluoroalkylation of tryptophan moieties.

Fluoroalkyl radicals are electrophilic and thus easily reacted with aromatic, especially heteroaromatic compounds [85]. Using 5,10,15,20-tetrakis(4-trimethylammoniophenyl)porphyrin tetra(*p*-toluenesulfonate) (TTMAPP, **81**, 1 mol %) or helical *N*′-di-*n*-propyl-1,13-dimethoxyquinacridinium tetrafluoroborate (*n*-Pr-DMQA^+^, **55**, 2.5 mol %) fluoroalkyl radicals are produced from the corresponding precursors **82** (Scheme 20) [86]. They have been added to a variety of tryptophan-containing peptides **83** and the resulting products **84** have been obtained with yields up to 74%. Using a corresponding terminal diiodide, two tryptophan moieties have been connected by a fluorinated linker. Linear peptides with two tryptophan units can be cyclized using similar diiodofluoroalkyl linkers. The fluoroalkyl iodides **85** carrying a biotin moiety have been used in this reaction to label enzymes such as carbonic anhydrase, albumin, phosphorylase, or bovine serum albumin. Biotinylation with this method was also carried out in living cells.

**Figure 16:**
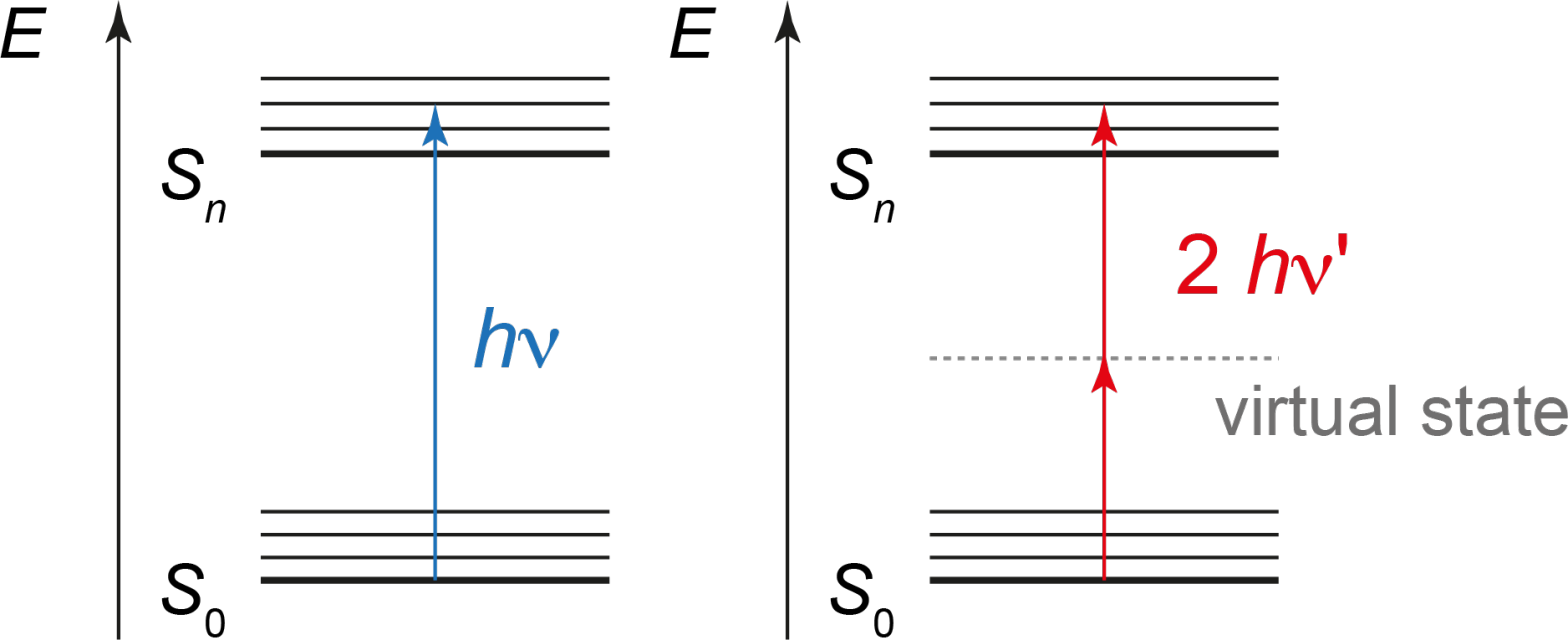
Simultaneous absorption of two photons of infrared light of low energy enables electronic excitation of compounds absorbing single photons in the visible spectrum domain.

Two-photon absorption is a very elegant method to use near infrared light for electronic excitation of molecules absorbing single photons in the visible domain of the light spectrum (Figure 16). The efficiency of such an excitation depends on the symmetry properties of the chromophore [87–88]. A two-photon excitation is carried out with corresponding lasers. This technique enables the usage of a larger variety of chromophores in medicinal and biochemistry and related domains while conserving the property of a relatively high tissue penetration [89–90]. Electronic excitation by two-photon absorption in tissues can be highly localized as it occurs only in the focal point of lensing. Thus, even a single cell in a tissue can be selectively addressed [91].

**Scheme 21:**
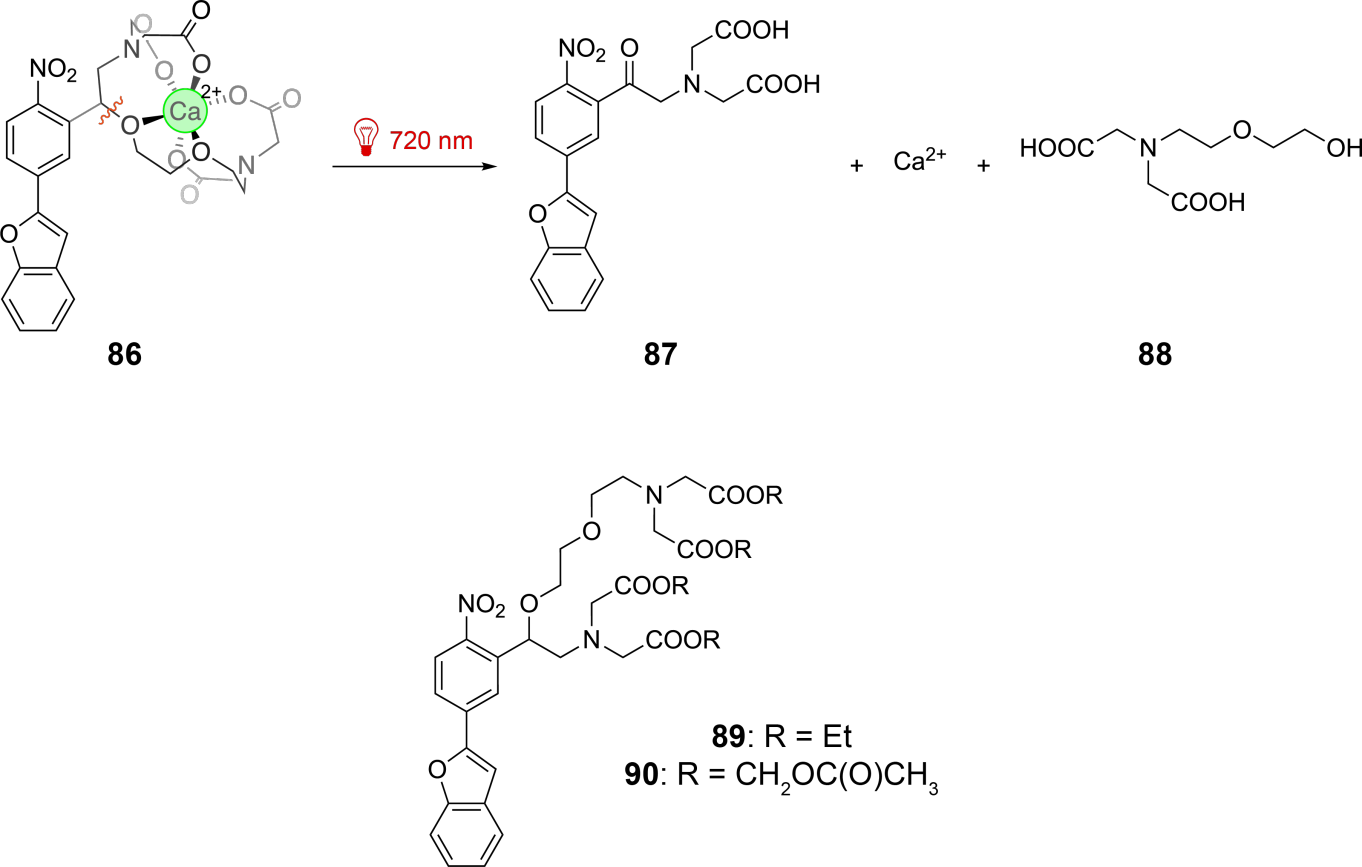
Uncaging Ca^2+^ ions using two-photon excitation with near infrared light.

Calcium ions play an important role in many biological phenomena, for example in neurotransmission. Calcium ions can efficiently be caged by ethylene glycol tetraacetic acid (EGTA) derivatives [92]. The cage was bound to a nitrophenyl benzofuran chromophore **86** (Scheme 21) [93]. This chromophore is considered as styrene derivative and such compounds possess a relatively high cross section for two-photon absorption. After absorption of two near infrared photons, the benzyl ether bond is cleaved and the cage is split into two fragments **87** and **88**, thus releasing Ca^2+^ ions. The cross section for this fragmentation is 20.7 GM at λ = 740 nm determined with the corresponding esters **89**. The cage was applied to whole-cell-clamped neurons as derivative **90**. By releasing the carboxylic acid protecting group, Ca^2+^ is complexed (**86**). The presynaptic terminals were stimulated by the uncaging two-photon absorption process and the influence on the postsynaptic currents was measured.

## Conclusion

Red-light photocatalysis has emerged as a powerful tool in both synthetic chemistry and biological applications, owing to its unique ability to drive reactions under mild, energy-efficient conditions. In synthetic chemistry, metal-based photocatalysts, particularly those involving heavy metals like ruthenium and osmium, have been instrumental in expanding the scope of red-light-driven photoredox transformations. These complexes are valued for their exceptional photophysical properties which have facilitated a range of efficient and scalable transformations. At the same time, the use of ligands such as phthalocyanines has opened new avenues by enabling the application of more abundant metals, such as zinc, copper, and cobalt, thereby promoting the development of more sustainable photocatalytic systems. In parallel, organic photocatalysts such as squaraines and cyanins have gained prominence as versatile and sustainable alternatives. These systems offer unique advantages in terms of tunability and environmental impact. Their strong absorption in the red and near-infrared (NIR) regions, combined with their ability to mediate both energy- and electron-transfer processes, underscores their potential for diverse synthetic applications. In biological contexts, the deep tissue penetration of red and NIR light enables precise and localized processes that are challenging under shorter wavelengths. Applications such as drug uncaging, the controlled release of nitric oxide, and the targeted generation of bioactive species highlight the transformative potential of red-light photocatalysis in medicine and biochemistry. However, significant challenges remain to fully exploit the potential of red-light photocatalysis. The design of photocatalysts with both high oxidation and reduction potentials in their excited states, as well as longer excited-state lifetimes, is critical for broadening the substrate scope and achieving greater efficiency. These advancements would enable access to more complex transformations and functionalization of substrates currently out of reach. Looking ahead, the coupling of red-light photocatalysis with organic electrochemistry through electrophotocatalysis offers exciting new perspectives. This innovative strategy could facilitate the generation of novel red-light-absorbing species with enhanced redox properties, enabling transformations that are inaccessible to either technique alone.

## Data Availability

Data sharing is not applicable as no new data was generated or analyzed in this study.
